# Targeting STING-induced immune evasion with nanoparticulate binary pharmacology improves tumor control in mice

**DOI:** 10.1172/JCI192397

**Published:** 2025-10-23

**Authors:** Fanchao Meng, Hengyan Zhu, Shuo Wu, Bohan Li, Xiaona Chen, Hangxiang Wang

**Affiliations:** 1 The First Affiliated Hospital, NHC Key Laboratory of Combined Multi-Organ Transplantation, Collaborative Innovation Center for Diagnosis and Treatment of Infectious Diseases, State Key Laboratory for Diagnosis and Treatment of Infectious Diseases, Zhejiang University School of Medicine, Zhejiang Province, Hangzhou, China.; 2 Jinan Microecological Biomedicine Shandong Laboratory, Jinan, Shandong Province, China.

**Keywords:** Immunology, Oncology, Cancer immunotherapy, Drug therapy, Nanotechnology

## Abstract

Harnessing the stimulator of IFN genes (STING) signaling pathway to trigger innate immune responses has shown remarkable promise in cancer immunotherapy; however, overwhelming resistance to intratumoral STING monotherapy has been witnessed in clinical trials, and the underlying mechanisms remain to be fully explored. Herein, we show that pharmacological STING activation following the intratumoral injection of a nonnucleotide STING agonist (i.e., MSA-2) resulted in apoptosis of the cytolytic T cells, IFN-mediated overexpression of indoleamine 2,3-dioxygenase 1 (IDO1), and evasion from immune surveillance. We leveraged a noncovalent chemical strategy for developing immunomodulatory binary nanoparticles (iBINP) that include both the STING agonist and an IDO1 inhibitor for treating immune-evasive tumors. This iBINP platform, developed by dual prodrug engineering and subsequent nanoparticle assembly, enabled tumor-restricted STING activation and IDO1 inhibition, achieving immune activation while mitigating immune tolerance. A systemic treatment of preclinical models of colorectal cancer with iBINP resulted in robust antitumor immune responses, reduced infiltration of Tregs, and enhanced activity of CD8^+^ T cells. Importantly, this platform exhibits great therapeutic efficacy by overcoming STING-induced immune evasion and controlling the progression of multiple tumor models. This study unveils the mechanisms by which STING monotherapy induces immunosuppression in the tumor microenvironment and provides a combinatorial strategy for advancing cancer immunotherapies.

## Abstract 2

This study unveils STING-induced immune evasion mechanisms through T-cell apoptosis and IDO1 overexpression. We developed an immunomodulatory nanoplatform combining a STING agonist and an IDO1 inhibitor to boost T-cell responses and reduce Treg infiltration, thereby proposing a combinatorial nanotherapy against immune-evasive cancers.

## Graphical abstract

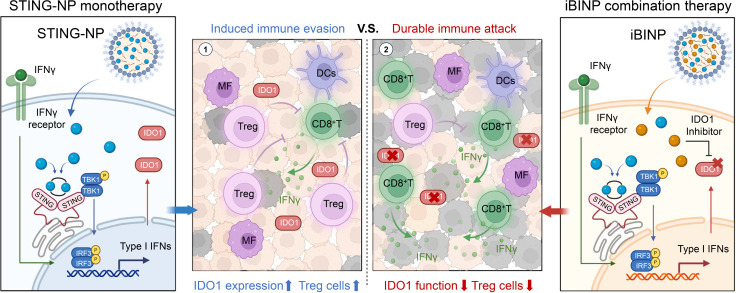

## Introduction

Immunotherapy, such as immune checkpoint blockade therapy, has achieved tremendous clinical success across various types of human cancer (1–4). However, many patients harbor “cold” tumors with an immunosuppressive tumor microenvironment (TME), which is a major obstacle for controlling tumor spread using immunotherapy (5–7). Agonists of the stimulator of IFN genes (STING) trigger inflammatory innate immune responses to potentially render the TME more conducive to immune activation (8, 9). Targeting STING-dependent signaling has been shown to elicit tumor antigen–specific adaptive immune responses and has spurred intensive interest in the development of clinically available STING therapies (10, 11). The intratumoral injection of STING agonists has shown great promise in preclinical cancer models and leads to an enhanced content of the tumor-intrinsic type I IFNs, increased C-X-C motif chemokine receptor 3–dependent (CXCR3-dependent) antitumor immunity, and increased survival (8). Despite these promising preclinical results, poor clinical outcomes and substantial therapeutic resistance have been witnessed in patients with cancer subjected to STING agonist monotherapy administered intratumorally (10, 11). For instance, a combination therapy of the first-in-class synthetic cyclic dinucleotides (CDNs) STING agonist, ADU-S100, and spartalizumab achieved a response rate of just 10.4% in phase I clinical trials (11). Given these predicaments associated with the clinical application of STING agonists, obtaining a better understanding of the mechanisms underlying STING therapy–induced immune evasion and devising therapeutic approaches are imperative for improving treatment responses.

Indoleamine 2,3-dioxygenase 1 (IDO1) catalyzes the conversion of the essential amino acid *L*-tryptophan (Trp) to the immunosuppressive metabolite *L*-kynurenine (Kyn), which plays a critical role in immunoregulation (12, 13). The mechanisms that contribute to the protumorigenic activities of IDO1-induced Trp starvation and the generated Kyn metabolites are suggested to trigger the apoptosis/dysfunction of effector T cells and the conversion of naive CD4^+^ T cells to FOXP3^+^ Tregs, thereby fostering an immunosuppressive TME that is favorable for tumor growth (14). In addition, the main Kyn metabolites are potent activators of the aryl hydrocarbon receptor that participates in the induction of apoptosis in immune cells, further dampening antitumor immunity (15). IDO1 is presented across multiple types of cancer, and its expression is upregulated by inflammatory cytokines such as IFN-γ (16, 17). Compelling evidence from preclinical and clinical studies substantiates that the STING agonist therapy–induced immune responses are evaded by several types of tumors via various immune-inhibitory molecules, with the cancer cells also exploiting the IDO1 pathway to avoid immunotherapy-mediated destruction (18–20). Therefore, the pharmacological targeting of the IDO1 pathway and Trp catabolism may represent a potential and hitherto underexplored approach for overcoming STING-induced immune evasion by tumors.

Herein, we show that the pharmacological activation of STING promotes the overexpression of the immunometabolic enzyme IDO1. A noncovalent chemical strategy that allows the overcoming of STING-induced immunosuppression has been delineated herein and relies on the use of a nanoparticle-based combination treatment with two pharmacological agents, MSA-2 (a STING agonist) and NLG919 (an IDO1 inhibitor). We show that this binary nanoparticle pharmacology scaffold not only mitigates the critical delivery and safety barriers of individual free drugs but also achieves high efficacy against multiple tumor models. Our findings provide key insights into STING-induced therapeutic resistance and carry important implications for the design of improved anticancer immunotherapies.

## Results

### MSA-2–induced activation of the STING pathway inflames the TME.

**Figure 1:**
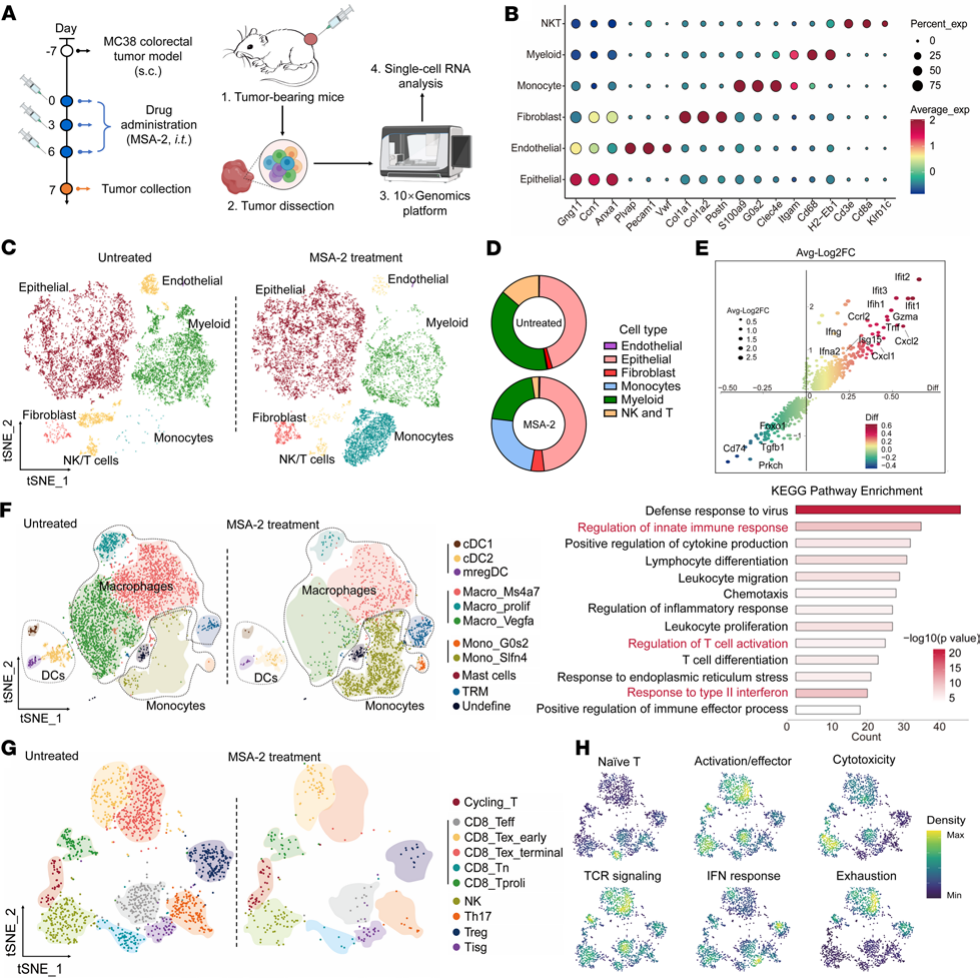
Intratumoral injection of MSA-2 elicits a potent proinflammatory response and causes immune cell death. (**A**) Schematic representation of single-cell transcriptome analysis. i.t., intratumoral, s.c., subcutaneous. Created with BioRender. (**B**) Dot plot showing marker gene expression across identified cell clusters. (**C**) The t-SNE plots of scRNA-seq data from the MC38 tumors. The suspended single cells were divided into 6 clusters, and each cluster was manually defined as a specific cell population. (**D**) The proportion of cells of each cluster in the tumor tissue obtained from the cohort. (**E**) Analysis of the expression of DEGs, showing up- and downregulated genes across all types of immune cells following MSA-2 treatment (top). KEGG enrichment analysis of DEGs after treatment with MSA-2 (bottom). (**F**) t-SNE plot of the subclusters of myeloid cells and monocytes. (**G**) t-SNE plot of the subclusters of NK and T cells. (**H**) Density plot of NK/T cells. The cells were functionally classified into 6 groups, including the naive, activated and effector, cytotoxic, TCR-signaling, IFN-responding, and exhausted T cells.

A comprehensive assessment of the responses of various immune cell phenotypes upon exposure to the STING agonist was performed using single-cell RNA sequencing (scRNA-seq) analysis on mouse MC38 subcutaneous tumors (Figure 1A). Following the intratumoral injection of MSA-2, a small-molecule STING agonist, large shifts were observed in the frequencies of numerous immune subsets in the TME; in particular, a notable increase in the numbers of monocytes was witnessed (Figure 1, B–D). MSA-2 also induced remarkable changes in the transcriptomic profile of several immune cell clusters. Most immune cells exhibited upregulated transcription of numerous proinflammatory genes (Figure 1E). Kyoto Encyclopedia of Genes and Genomes (KEGG) analysis revealed that these genes were predominantly enriched in immune response pathways and the numerous proinflammatory pathways associated with antitumor activity (Figure 1E). To obtain a better understanding of the changes in the immune landscape of TME, the myeloid cells and monocytes were stratified into subclusters (Figure 1F and Supplemental Figure 1A; supplemental material available online with this article; https://doi.org/10.1172/JCI192397DS1). Monocytes were observed to rapidly infiltrate the tumor following the in situ administration of MSA-2, suggesting the induction of strong inflammatory immune responses (Figure 1F). An increase in the numbers of other types of myeloid cells was not observed, whereas notable decreases were evident in the proportions of DCs (including the conventional type 1 and type 2 DCs) and proliferative macrophages (Supplemental Figure 1B); NK and T cells are subsets of the crucial lymphocytes that mediate cytotoxicity (21). The NK/T cells were therefore subdivided into 10 subclusters. While the frequency of cycling T cells increased, indicating the recruitment of T cells to the TME, the overall number of NK/T cells decreased following the intratumoral administration of MSA-2 (Figure 1G and Supplemental Figure 1C). These findings are consistent with previous observations that STING activation may induce T cell apoptosis (22, 23). Furthermore, these functional subsets of NK and T cells were classified into 6 groups according to their characteristic genes, as shown in Figure 1H. The results revealed that activated/effector T cells were almost eliminated following STING monotherapy, potentially impairing T cell–mediated antitumor responses (Figure 1H and Supplemental Figure 1D).

The cellular interactions between the DCs and T cells within the tumor tissues were subsequently investigated (Supplemental Figure 1, E–H). Cell-cell communication analysis revealed strong interactions between cDC1 and CD8^+^ T cells in the MSA-2–treated tumors that involve the IFN-β1 (IFNB1)/type-1 IFN receptor signaling pathway. Moreover, ligand-receptor pairs associated with chemotaxis and adhesion of the immune cells were predominantly concentrated. However, other T cell receptor (TCR) signaling pathways or inhibitory ligand-receptor communications were not observed in the group subjected to MSA-2 treatment, which may be attributable to the extensive apoptosis of immune cells.

### STING activation may induce Treg-mediated tumor immunosuppression.

**Figure 2:**
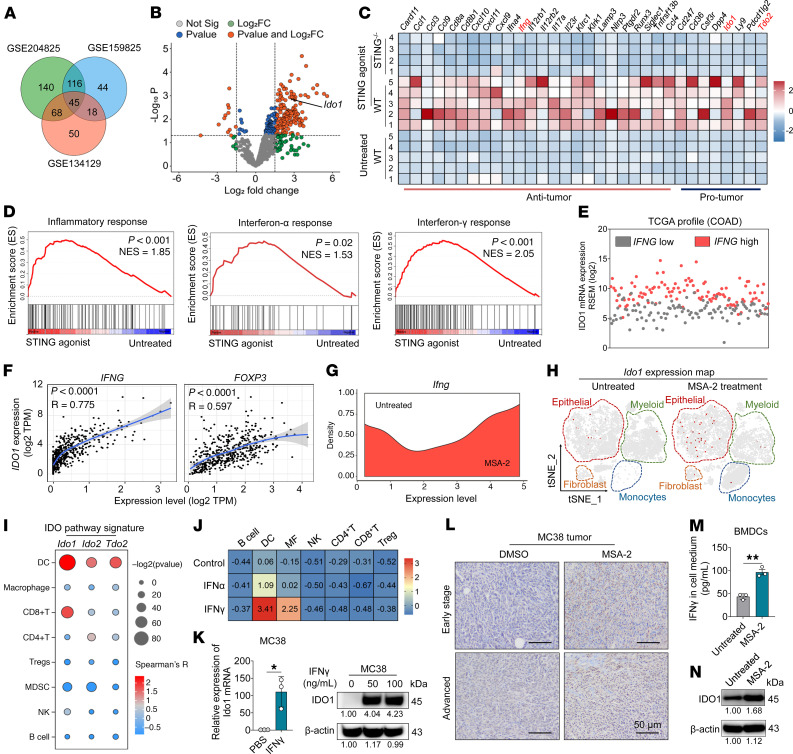
STING pathway activation upregulates the expression of *Ido1*. (**A**) Venn diagram showing the intersection of DEGs from the 3 GEO databases (GSE204825, GSE159825, and GSE134129). (**B**) Volcano plot illustrating changes in gene expression after the administration of STING agonist, with the *Ido1* gene marked by an arrow. (**C**) Heatmap of DEGs, excluding some genes with unclear functions. The DEGs were divided into 2 clusters of antitumor and protumor genes. (**D**) GSEA analysis demonstrating the enrichment of DEGs in various pathways. (**E** and **F**) Analysis of the correlation between the expression of *IDO1* and that of the genes (*IFNG* or *FOXP3* in patients with colon adenocarcinoma [COAD]) using the TCGA database. (**G**) Expression of *Ifng* across all types of immune cells. (**H**) Feature plot of *Ido1* showing inducible patterns of expression upon MSA-2 administration. Red indicates *Ido1*^hi^ cells. (**I**) Expression of signature genes of the IDO pathway (*Ido1*, *Ido2*, and *Tdo2*) in various immune cells. (**J**) Alterations in *Ido1* gene expression following stimulation with IFN-α and IFN-γ. (**K**) Quantitative real-time PCR analysis of *Ido1* and Western blot analysis of the protein IDO1 in IFN-γ–treated MC38 tumor cells (*n* = 3). β-Actin was used as internal control. (**L**) IHC staining of *Ido1* at the tumor site with or without MSA-2 administration. Early-stage and advanced tumors represent tumor volumes of approximately 500 and 1,500 mm^3^, respectively. Scale bar: 50 μm. (**M**) IFN-γ secretion was assessed using ELISA (*n* = 3). (**N**) Western blot analysis of IDO1 protein expression in MSA-2–treated or untreated BMDCs. Data are depicted as the mean ± SD. *P < 0.05, **P < 0.01, as determined by Student’s *t* test.

The expression of immune-related genes in the control and STING agonist–treated tumor groups was analyzed utilizing data from the GEO databases (accession GSE134129, GSE159825, and GSE204825). A total of 45 overlapping genes that were predominantly upregulated upon STING activation were identified (Figure 2A). Volcano plot analysis corroborated that most of these genes were upregulated upon the activation of the STING pathway (Figure 2B). Gene Ontology enrichment and KEGG analyses identified the enrichment of the differentially expressed genes (DEGs) in pathways associated with immune activation (Supplemental Figure 2A). Notably, the STING agonists augmented the expression of antitumorigenic immune-related genes (24, 25), including *Cxcl9*, *Cxcl10*, and *Ifng*, while concurrently inducing the expression of protumorigenic genes such as *Cd36*, *Ido1*, and *Tdo2* in WT mice (16). The diminished therapeutic efficacy in STING-deficient mice substantiated the dependency of these changes in gene expression on the activation of the STING pathway (Figure 2C). Moreover, gene set enrichment analysis (GSEA) revealed primary enrichment of transcriptional signatures associated with inflammatory responses, type I IFN signaling, and IFN-γ–mediated responses (Figure 2D). *Ido1* was identified as an oncogene whose transcription was activated by the STING agonists and encodes the Trp catabolic enzyme IDO1, which is a critical regulator within the immune-tolerant TME (20). The Cancer Genome Atlas (TCGA) database revealed that patients with colon cancer exhibiting high *IFNG* expression were more likely to have elevated expression of *IDO1* (Figure 2E). A positive correlation between the expression of *IDO1* and that of *IFNG* and *FOXP3* in the human transcriptome was also observed (Figure 2F). The results of scRNA-seq analysis revealed that *Ifng* transcription increased in a pronounced manner (Figure 2G). Despite these profound results, MSA-2 monotherapy induced the upregulation of *Ido1* transcription, as evidenced by t-SNE dimensionality feature plots (Figure 2H), which aligns with prior GEO database analysis.

The signature markers of the IDO pathway, including *Ido1*, *Ido2*, and *Tdo2*, were further analyzed the immune cells using TIMER2.0 (http://timer.cistrome.org/) (26), which revealed the high expression of *Ido1* in DCs and CD8^+^ T cells (Figure 2I). Following stimulation with proinflammatory cytokines such as IFN-α and IFN-γ, an upregulated expression of *Ido1* was witnessed in the DCs and macrophages (GEO dataset, accession GSE112876, Figure 2J), which is consistent with the previous finding that inflammatory cytokines such as IFN-γ induce the overexpression of *IDO1* (27). In addition, an experimental validation was also carried out; the exogenous addition of recombinant IFN-γ (50 ng/mL) was sufficient to induce a significant upregulation of *Ido1* at both transcriptional and translational levels in MC38 tumor cells (Figure 2K). Immunohistochemistry (IHC) analysis of the MC38 tumor tissues showed that MSA-2 exposure led to higher IDO1 expression in both early-stage and advanced tumors compared with that in the untreated tumors (Figure 2L). Moreover, MSA-2 treatment was found to trigger the abundant secretion of IFN-γ, which in turn increased IDO1 expression in the BMDCs, supporting the IFN-γ–IDO axis (Figure 2, M and N, and Supplemental Figure *2*B). Collectively, these results suggest that IDO1 is upregulated upon the MSA-2–induced activation of the STING pathway, with this transcriptional activation likely to occur in both tumor and immune cells.

### Assembly of STING-activating and IDO-inhibitory prodrugs into immunomodulatory binary nanoparticles.

**Figure 3:**
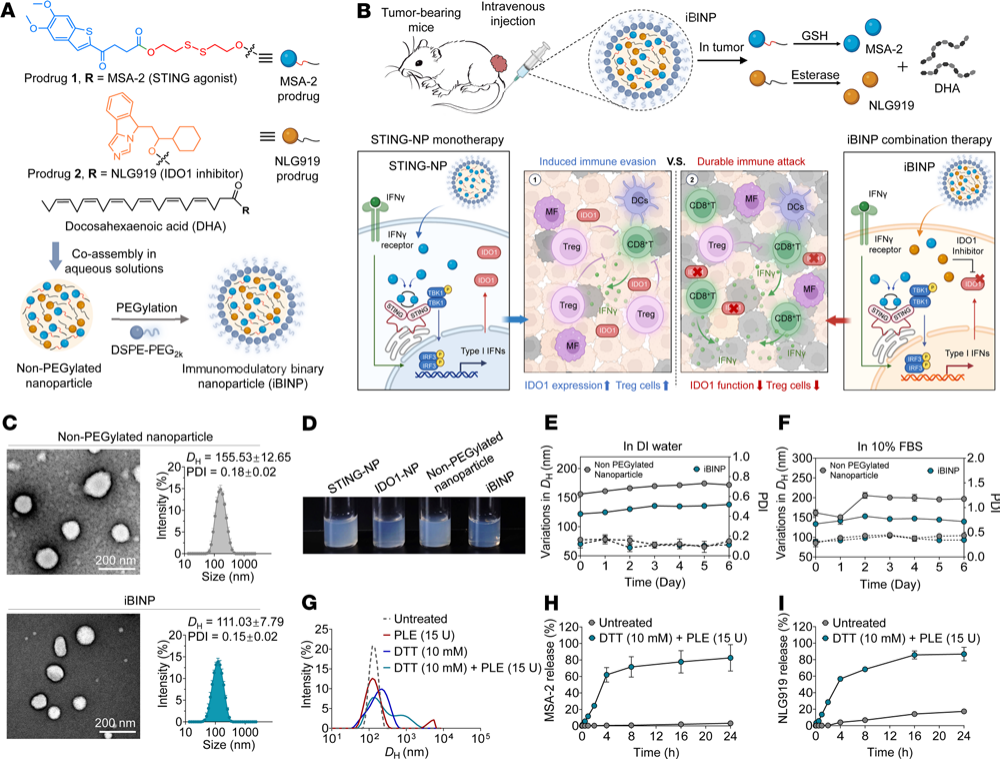
Preparation and characterization of the iBINP. (**A**) Structural formula and schematic representation of the synthesis route of the STING agonist MSA-2 and the IDO1 inhibitor NLG919. (**B**) Diagrammatic representation of the dual prodrug regimen. Created with BioRender. (**C**) Electron microscopy imaging and size distribution plot of co-loaded nanoparticle self-assemblies (*n* = 3). Scale bar: 200 nm. (**D**) Visual appearance of the solutions of the various nanoparticles. STING-NP and IDO1-NP represent MSA-2 and NLG919 prodrugs assembled in aqueous solutions, respectively. Non-PEGylated nanoparticle and iBINP represent coloaded nanoparticle assemblies in the absence or presence of DSPE-PEG_2000_, respectively. Evaluation of the stability of nanoparticles in the presence or absence of DSPE-PEG_2000_ in (**E**) DI water or (**F**) 10% FBS by monitoring changes in particle size and PDI (*n* = 3). (**G**) Changes in particle size of nanoparticle assemblies under conditions that simulate various in vivo TME. Drug release profiles of (**H**) MSA-2 and (**I**) NLG919 prodrugs (*n* = 3). Data are presented as mean ± SD.

Building on these findings, STING agonist monotherapy was anticipated to inadvertently upregulate IDO1 and potentially, prompt to tumor escape, despite provoking robust tumor-specific immune responses. We hypothesized that this therapeutic resistance can be mitigated via a combination therapy of the STING agonist MSA-2, whose functioning requires spontaneous intracellular activation, and the IDO1 inhibitor NLG919 (Figure 3A). As a proof of principle, the docosahexaenoic acid (DHA, an omega-3 fatty acid) promoiety was selected for esterifying the water-insoluble compounds MSA-2 and NLG919. The resulting prodrugs, when attached with polyunsaturated fatty acids via ester bonds, are capable of forming nanoassemblies in aqueous solutions. A disulfide linker was designed for synthesis of the MSA-2–DHA ligate and can be spontaneously cleaved under the reducing conditions encountered in the TME (Figure 3B). Additionally, we hypothesized that MSA-2 treatment, while capable of exerting beneficial effects with respect to immune activation, may also trigger the activation of the IDO pathway to ultimately induce immune tolerance. By contrast, the immunomodulatory binary nanoparticles (iBINP) generated herein activated the innate immune pathways while concurrently preventing the onset of immune tolerance via the synergistic effects of their components, the STING agonist MSA-2, and the IDO1 inhibitor NLG919 (Figure 3B). The structures of the prodrugs 1 (MSA-SS-DHA) and 2 (NLG919-DHA) were confirmed using ¹H NMR spectroscopy (Supplemental Figure 3, A and B).

To aid the aqueous self-assembly of MSA-2 and NLG919 prodrugs, both conjugates were dissolved in DMSO and subsequently mixed with deionized (DI) water under ultrasonication, resulting in the formation of stable nanosuspensions. The colloidal stability was further increased by PEGylating the surface of these suspensions with clinical use–certified 1,2-distearoyl-*sn*-glycero-3-phosphoethanolamine-*N*-(methoxy [polyethylene glycol] 2000) (DSPE-PEG_2000_) at a low-weight percentage (e.g., 10 %). Transmission electron microscopy and dynamic light scattering (DLS) analyses revealed spherical nanoparticles and a hydrodynamic diameter (*D*_H_) of approximately 150 nm for the bare non-PEGylated binary nanoparticles (Figure 3C). With the addition of the amphiphilic DSPE-PEG_2000_ matrix, the mean *D*_H_ of the iBINP was refined to approximately 110 nm with a lower polydispersity index (PDI). The iBINP solution appeared more transparent compared with that of other non-PEGylated nanoparticles (Figure 3D). Further analysis showed that these nanoparticles were stable in PBS, but only iBINP remained stable in PBS containing 10% FBS, suggesting that the reduction of surface hydrophobicity using the PEGylation matrix aided the stabilization of the formulations (Figure 3, E and F). DLS was then employed for examining variation in the sizes of these nanoparticle assemblies in response to dithiothreitol (DTT) and/or pig liver esterase (PLE), given the responsive linker chemistry (Figure 3G). The formation of large aggregates in the iBINP solution following 12 hours of incubation with DTT/PLE can be attributed to dual-responsive prodrug hydrolysis, which disrupts the nanostructures. Dialysis against PBS (pH 7.4) at 37°C in the presence or absence of DTT/PLE allowed the evaluation of drug release from the iBINP (Figure 3, H and I). Minimal release of MSA-2 and NLG919 was observed after 24 hours of dialysis against PBS. By contrast, drastically accelerated release kinetics was observed in the presence of DTT/PLE, thereby validating the stability of the iBINP under physiological conditions and the dual-responsive drug-releasing feature. Furthermore, it was efficiently internalized by both tumor and immune cells, followed by successful lysosomal escape (Supplemental Figure 3C).

### iBINP enhances immune response via the pharmacological modulation of IDO signaling in vitro.

**Figure 4:**
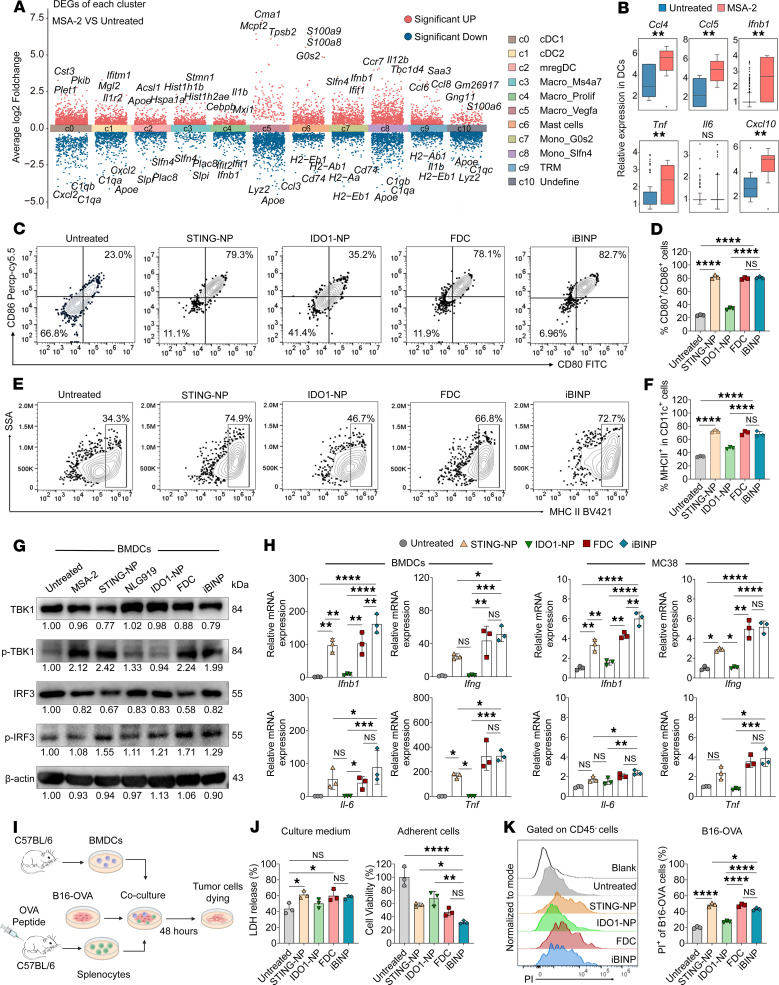
Combination therapy with STING agonist and IDO1 inhibitor improves antitumor immunotherapy under in vitro conditions. (**A**) Violin plot showing differentially expressed genes (DEGs) in myeloid and monocyte clusters from MC38 tumors after MSA-2 treatment, based on scRNA-seq analysis. (**B**) Relative expression of key cytokine and chemokine genes in dendritic cells (DCs) after MSA-2 treatment. (**C**–**E**) Flow cytometric analysis of DC maturation. Representative plots and quantification of the costimulatory molecules CD80/CD86 (**C** and **D**) and MHC class II (**E** and **F**) on bone marrow–derived dendritic cells (BMDCs) after various treatments (*n* = 3). (**G**) Western blot analysis evaluating the phosphorylation status of proteins associated with the STING pathway. (**H**) Quantitative real-time PCR (qRT-PCR) analysis of inflammation-associated genes (*Ifnb1*, *Ifng*, *Il6*, and *Tnf*) in BMDCs subjected to different treatments (*n* = 3). (**I**) Schematic representation of the in vitro validation of drug-enhanced cytotoxicity mediated by antigen-specific CD8^+^ T cells. Created with BioRender. (**J**) Evaluation of cytotoxicity using LDH assay and CCK8 assay for evaluating the viability of tumor cells (*n* = 3). (**K**) Flow cytometric analysis of specific cell death, showing the percentage of PI^+^ cells in the target B16-OVA population (*n* = 3). Data are presented as the mean ± SD. *P < 0.05, **P < 0.01, ***P < 0.001, ****P < 0.0001. One-way ANOVA was employed for the evaluation, with Tukey’s correction for multiple comparisons.

Preliminary experiments revealed that intratumorally delivered MSA-2 led to the recruitment of monocytes to the tumor site, which was accompanied by activation of the transcription of inflammation-related genes, as depicted in a volcano plot (Figure 4A). Notably, monocytes also function as precursor cells that further differentiate into mature antigen-presenting cells (APCs) such as DCs and macrophages (28). DCs play an essential role in antitumor immunity and are the primary target cells for STING agonists (29, 30). Indeed, MSA-2 treatment was found to activate the STING pathway in DCs, as manifested by the transcriptional activation of the type I IFN (IFN-β) and numerous inflammatory chemokines and cytokines (Figure 4B). We further sought to examine whether the nanoparticles retained their activity as compared with the free drug form by performing an ELISA. STING-NP induced the release of IFN-β from BMDCs to a similar extent as free MSA-2, confirming the potent and spontaneous activation of STING signaling (Supplemental Figure 4A). Furthermore, analysis of the Kyn/Trp ratios in both BMDCs and MC38 cells demonstrated that IDO1-NP inhibited Trp metabolism as effectively as an equivalent concentration of free NLG919, supporting its sufficient enzymatic inhibition (Supplemental Figure 4B). To clarify whether combined treatment with a STING agonist and IDO inhibitor impeded the maturation of DCs, the BMDCs isolated from C57 mice were treated with various drug formulations, and the expression of costimulatory markers CD80/CD86 (Figure 4, C and D) and MHC-II (Figure 4, E and F) was analyzed using flow cytometry. The results revealed that STING agonist–formulated nanoparticles promoted DC maturation, which was not reversed by the addition of the IDO inhibitor NLG919, suggesting that the iBINP platform can notably potentiate STING agonist–mediated antitumor responses without compromising its ability to promote the maturation of DCs.

STING activation following iBINP treatment was verified by evaluating the phosphorylation status of the key signaling proteins TANK-binding kinase 1 (TBK1) and IFN regulatory factor 3 (IRF3) (Figure 4G). Consistent with the results of Western blot analysis, the transcriptional activation of *Ido1* and various proinflammatory factors, such as *Ifnb1*, *Ifng*, *Il6*, and *Tnf*, was verified in BMDCs and MC38 tumor cells treated with STING-NP, free drug combination (FDC), and iBINP compared with those of the negative control group (Figure 4H and Supplemental Figure 4, C and D). Notably, the upregulation of these inflammatory factors and *Ido1* was more pronounced in immune cells than in both human and murine cancer cells, suggesting that immune cells are the primary responders to the STING agonists in this context. In vitro experiments involving specific cytotoxic T lymphocyte–induced (CTL-induced) killing of cancer cells were employed for assessing the iBINP-induced immune response. For this purpose, splenocytes were isolated from mice, pulsed twice with ovalbumin (OVA) peptide 257–264, and then cocultured with the drug regimen–treated BMDCs and B16F10-OVA cells to elicit an OVA-specific CTL response (Figure 4I). Cell death was evaluated by assaying for lactate dehydrogenase (LDH) activity, which revealed that a combination of NLG919 (in any pharmaceutical form such as free drug or nanoparticles) with the other drugs did not attenuate DC maturation and the subsequent cytotoxicity mediated by OVA-specific T cells. Tumor cell death was substantiated using cell counting kit-8 (CCK8) assays, and further microscopic analysis revealed pronounced adhesion of the CTLs to B16F10-OVA cells (Figure 4J and Supplemental Figure 4E). To further verify whether the tumor cells were dying, we specifically labeled a CD45^–^ tumor cell subset using flow cytometry (Supplemental Figure 4F) and found that STING-NP and iBINP treatments notably increased T cell–triggered killing of tumor cells, as shown by propidium iodide^+^ staining (Figure 4K). These assays demonstrate the robust capacity of STING-NP, the FDC, and iBINP for triggering an immune response, culminating in effective T cell–mediated destruction of tumor cells. Overall, the findings confirm the immune activation potential of iBINP under in vitro conditions.

### Nanoparticle delivery facilitates intratumoral and lymphatic accumulation.

**Figure 5:**
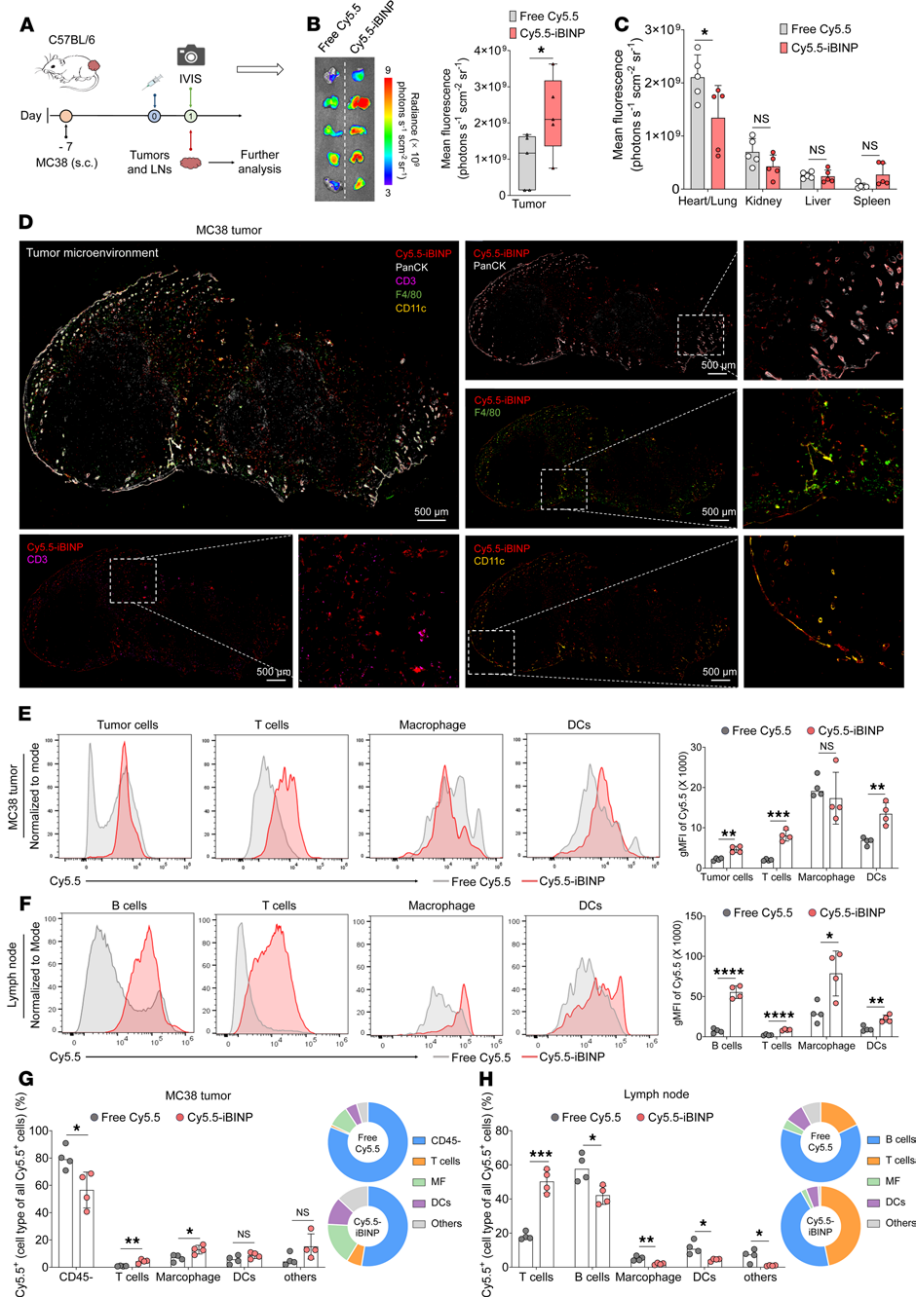
iBINP enhances tumor accumulation and lymphatic delivery, promoting immune cell targeting. (**A**) Schematic of the experimental design for evaluating the biodistribution of Cy5.5-labeled nanoparticles in mice bearing subcutaneous MC38 tumors. (**B**) Ex vivo fluorescence imaging showing tumor accumulation of Cy5.5-labeled nanoparticles (Cy5.5-iBINP) compared with free Cy5.5 dye. The bar chart shows the quantification of mean fluorescence intensity in the tumors (*n* = 5). (**C**) Ex vivo fluorescence quantification in major organs shows the biodistribution profile of the nanoplatform compared with free Cy5.5 dye (*n* = 5). (**D**) Immunofluorescence images of a MC38 tumor section after injection, showing the spatial distribution of tumor cells (PanCK, white), T cells (CD3, violet), macrophages (F4/80, green), and DCs (CD11c, orange). Scale bar: 500 μm. (**E** and **F**) Flow cytometric analysis of nanoparticle uptake by different cell populations within MC38 tumors (**E**) or TDLNs (**F**). (**G** and **H**) Quantification of the cellular distribution of Cy5.5^+^ cells within the MC38 tumor (**G**) or TDLNs (**H**) (*n* = 4). The bar chart and corresponding pie charts show the percentage of total Cy5.5^+^ cells in specific type of cells. Data are presented as mean ± SD. Statistical analysis by Student’s *t* test (**B**, **C**, **E**, and **F**). *P < 0.05, **P < 0.01, ***P < 0.001.

To investigate the in vivo behavior of the iBINP platform, we intravenously administered near-infrared (NIR) dye Cy5.5-labeled iBINP (Cy5.5-iBINP) or free Cy5.5 dye into MC38 tumor-bearing mice (Figure 5A). In vivo and ex vivo imaging showed that nanoparticle-mediated delivery increased fluorescence signals in tumors compared with free Cy5.5 administration (Figure 5B and Supplemental Figure 5A). Analysis of tissue biodistribution affirmed no obvious differences in NIR signals between free Cy5.5 and Cy5.5-iBINP administered mice (Figure 5C and Supplemental Figure 5B). To further unravel whether iBINP can extravasate into tumors and interact with immune cells following intratumoral accumulation, we conducted immunofluorescence staining of tumor sections. iBINP was found to deeply penetrated into the tumor parenchyma, where these particles colocalized well with tumor cells (PanCK), macrophages (F4/80), T cells (CD3), and DCs (CD11c) (Figure 5D and Supplemental Figure 5C). To quantify this distribution, we performed flow cytometry on excised tumors and tumor-draining lymph nodes (TDLNs; Supplemental Figure 5, D and E). In both tissues, administration of iBINP resulted in higher uptake across all major cell types, particularly in macrophages and DCs, compared with free Cy5.5 administration (Figure 5, E and F). Analysis of the composition of all Cy5.5^+^ cells revealed that while tumor cells (CD45^–^) constituted a large fraction of uptake, nanoparticles were disproportionately enriched in macrophages and DCs relative to T cells (Figure 5, G and H). These results suggest that the iBINP delivery platform can target tumors and TDLNs, where it is preferentially internalized by APCs.

### iBINP overcomes therapeutic resistance and induces durable antitumor immunity.

**Figure 6:**
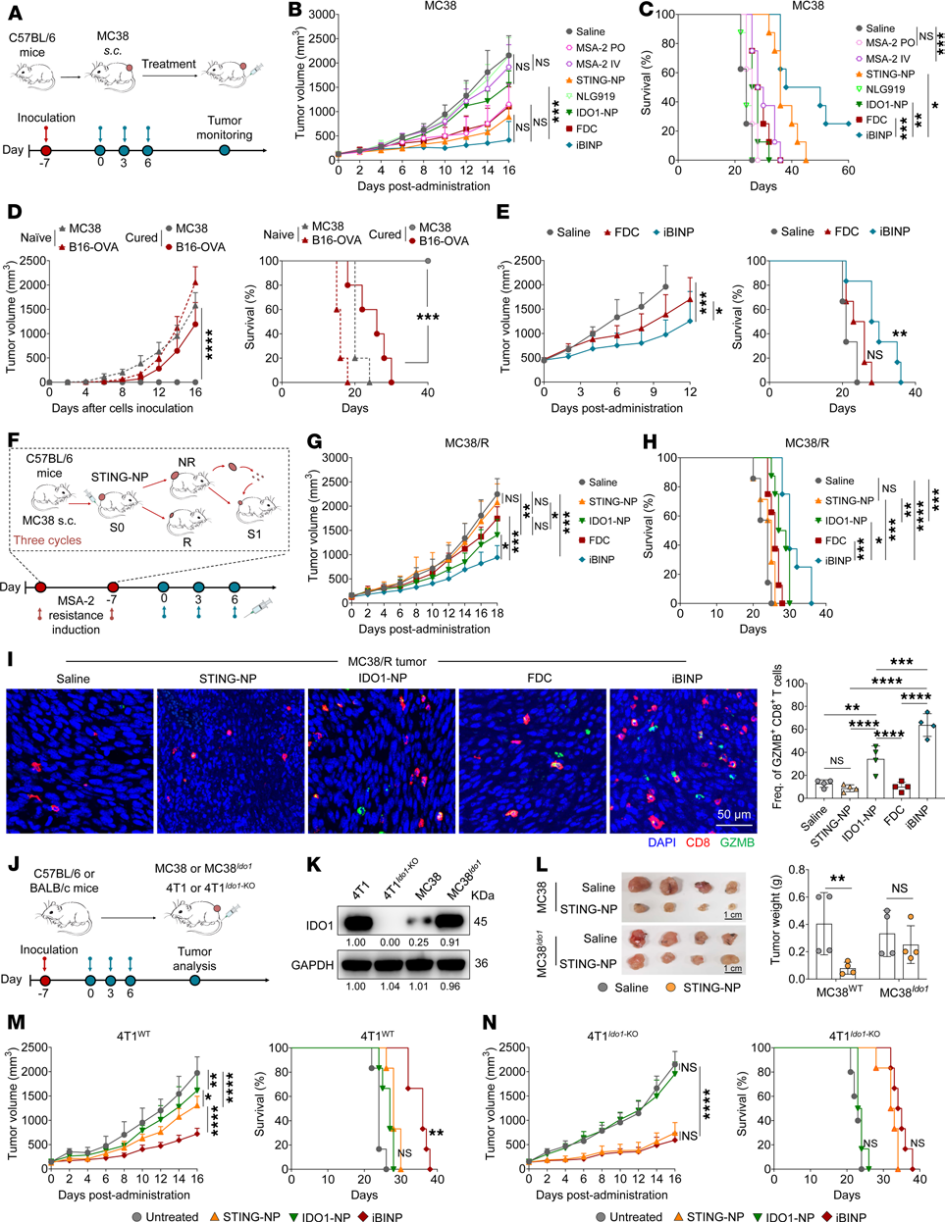
iBINP overcomes acquired resistance to STING monotherapy by targeting the IDO1 feedback loop. (**A**) Schematic representation of the experimental protocol employed for the MC38 subcutaneous tumor model. (**B**) Tumor growth kinetics and (**C**) survival analysis of MC38 tumors treated with different formulations (*n* = 8 mice/group). (**D**) Tumor rechallenge experiment in iBINP-cured mice (*n* = 5 mice/group). (**E**) Efficacy of iBINP in a large, established MC38 tumor model, showing tumor growth and survival (*n* = 6 mice/group). (**F**) Schematic illustrating the in vivo generation of a STING agonist–resistant MC38 model (MC38/R). (**G**) Tumor growth curves of mice bearing the established MC38/R tumors, following treatment with saline, STING-NP, IDO1-NP, FDC, or iBINP. (**H**) Survival analysis of the MC38/R tumor-bearing mice after treatments (*n* = 7 for saline and STING-NP; *n* = 8 for other groups). (**I**) Representative images show staining for CD8^+^ T cells (green), granzyme B (GZMB, red), and nuclei (DAPI, blue). The bar chart shows the quantification of the frequency of GZMB^+^CD8^+^ T cells (*n* = 4). Representative source images for the analysis are shown in Supplemental Figure 6F. Scale bar: 50 μm. (**J**) Schematic of the experiment designed to test therapy response in MC38^Ido1^ and 4T1*^Ido1-KO^* tumors. (**K**) Western blot analysis verifying the protein expression of *IDO1* in MC38^Ido1^, MC38, 4T1, and 4T1*^Ido1-KO^* cell lines. (**L**) Representative images of excised tumors and a bar chart showing the tumor weight from mice treated with saline or STING-NP (*n* = 4). Scale bar: 1 cm. (**M** and **N**) Comparison of therapeutic efficacy in WT versus Ido1-knockout 4T1 tumor models. Shown are the tumor growth curves and corresponding survival analyses for mice bearing 4T1*^WT^* tumors (**M**) or 4T1*^Ido1-KO^* tumors (**N**) after treatment with the indicated formulations (*n* = 5–6 mice/group). Data are presented as mean ± SD. Statistical analysis by 2-way ANOVA with Turkey’s multiple comparisons test (**B**, **D**, **E**, **G**, **M**, and **N**), log-rank test (**C**–**E**, **H**, **M**, and **N**), 1-way ANOVA with Turkey’s multiple comparisons test (**I**), or Student’s *t* test (**L**). *P < 0.05, **P < 0.01, ***P < 0.001, ****P < 0.0001.

The efficacy of the codelivered iBINP platform was evaluated in a mouse model of CRC bearing naive MC38 syngeneic tumors. The intravenous administration every 3 days was initiated when the tumors attained volumes of approximately 100 mm^3^ (Figure 6A). In contrast to the partial antitumor response observed with STING-NP treatment, sustained inhibition and regression of the tumors were witnessed in the iBINP-treated mice. This discrepancy is attributable to IDO1 inhibition that reversed the immunosuppressive TME (Figure 6, B and C). Transient weight loss was observed in the mice receiving intravenous free MSA-2 (Supplemental Figure 6A). In stark contrast, all nanoparticle treatments were well tolerated. To probe whether iBINP treatment regimen induced a long-term immunological memory, mice with complete regression of MC38 tumors were rechallenged with subcutaneous MC38 or irrelevant B16-OVA implantation. The cured mice completely rejected the emergence of MC38 tumors but not were sufficient to control B16-OVA tumor growth, indicating a durable tumor-specific immune memory (Figure 6D). Furthermore, we evaluated the efficacy of iBINP in a more clinically relevant, large tumor model (i.e., treatment was initiated at ~500 mm^3^ in volume). iBINP delayed tumor growth and extended mouse life span compared with the FDC treatment, supporting its potential for treating advanced tumors (Figure 6E).

To further highlight the superiority of the binary system developed herein, a STING therapy–resistant tumor model was established in mice. The mice were administered STING-NP for 3 cycles to develop the STING therapy–resistant MC38 subcutaneous tumor model (MC38/R; Figure 6F). Monitoring of tumor growth kinetics and overall survival of the mice verified the resistance of MC38/R tumors to STING therapy (Supplemental Figure 6, B and C). Furthermore, IHC analysis revealed a progressive increase in IDO1 expression and the numbers of Tregs in the third generation of tumors (S3), which is indicative of tumor immune evasion and the potentially suboptimal efficacy of STING agonists (Supplemental Figure 6D). Despite an aggressive tumor burden, the iBINP therapy continued to exert robust tumor-suppressive effects, extending the survival of mice bearing immunosuppressive MC38/R tumors (Figure 6, G and H) without causing any loss of body weight (Supplemental Figure 6E). Immunofluorescence staining of the tumor sections for CD8a and granzyme B (GZMB) revealed that codelivered immunomodulatory agents (iBINP) elicited a potent cytotoxic CD8^+^ T cell response (Figure 6I and Supplemental Figure 6F). In addition, immunohistochemical staining for FOXP3 revealed that STING agonist monotherapy led to an increase in intratumoral Tregs. In contrast, all treatments containing the IDO1 inhibitor, including IDO1-NP and iBINP, reduced the infiltration of these immunosuppressive cells (Supplemental Figure 6G).

To explore the role of IDO1 in mediating resistance, we thus established IDO1-overexpressing MC38 tumors (Supplemental Figure 6H), and, in parallel, we included an *Ido1*-knockout cancer model to examine the effects of our binary design rationale (Figure 6, J and K). Compared with naive MC38 tumors, STING-NP monotherapy was largely ineffective in the IDO1-enforced model, indicating that high IDO1 confers STING therapy resistance (Figure 6L). In the WT 4T1 breast cancer model with high IDO1 expression, the efficacy of STING-NP monotherapy was attenuated and lower than combinatory iBINP therapy (Figure 6M). In contrast, when *Ido1* was knocked out from the tumor cells (4T1*^Ido1-KO^*), the activity of STING-NP monotherapy was recovered, showing similar tumor growth inhibition to iBINP treatment (Figure 6N and Supplemental Figure 6I). Taken together, these results confirmed that the nanotherapeutic strategy using iBINP is potentially capable of overcoming STING-induced therapeutic resistance and immune evasion, thereby highlighting the rational design for priming immune responses.

### Remodeling of TME by iBINP.

**Figure 7:**
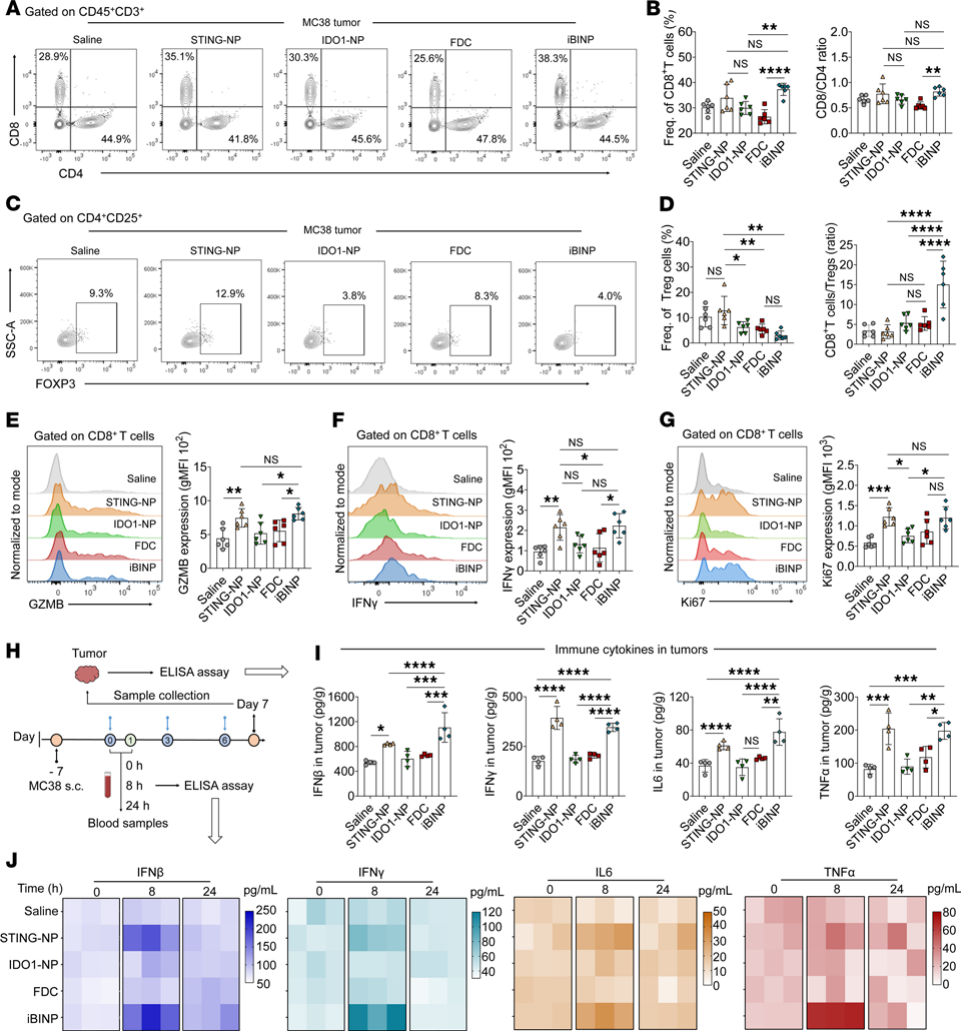
iBINP reshapes the tumor immune microenvironment by reducing Tregs to unleash cytotoxic T cell potential. (**A** and **B**) Flow cytometric analysis of tumor-infiltrating T lymphocytes (TILs) in MC38 tumors. (**A**) Representative plots show CD4^+^ and CD8^+^ populations gated on live CD45^+^CD3^+^ cells. (**B**) Quantification shows the frequency of CD8^+^ T cells and the CD8^+^/CD4^+^ ratio (*n* = 6). (**C** and **D**) Analysis of intratumoral Tregs. (**C**) Representative plots and (**D**) quantification of Treg frequency and the CD8^+^/Tregs ratio. (**E**–**G**) Functional analysis of tumor-infiltrating CD8^+^ T cells (*n* = 6). Representative histograms and quantification of the geometric mean fluorescence intensity (gMFI) for the cytotoxic effector molecule GZMB (**E**), IFN-γ (**F**), and the proliferation marker Ki67 (**G**) (*n* = 6). (**H**) A schematic of the sample collection timeline is shown. (**I**) Evaluation of the release of the proinflammatory cytokines IFN-β, IFN-γ, IL-6, and TNF-α using ELISA (*n* = 4). (**J**) Evaluation of the secretion of IFN-β, IFN-γ, IL-6, and TNF-α in serum samples from mice of the various groups using ELISA at 0, 8, and 24 hours after administration (*n* = 3). Data are presented as the mean ± SD. *P < 0.05, **P < 0.01, ***P < 0.001, ****P < 0.0001. One-way ANOVA along with Tukey’s multiple comparison test were employed.

The impact of codelivering the STING agonist and IDO1 inhibitor on the immune system was evaluated by analyzing the immune cell phenotypes in the tumor tissues and immune organs (Supplemental Figure 7, A and B). Notably, the combination therapy with iBINP resulted in T cell expansion, particularly that of the CD8^+^ T cells, within the tumor-infiltrating CD3^+^ T cell population (Figure 7A and Supplemental Figure 7C). The increased proportion of CD8^+^/CD4^+^ cells in mice treated with STING-NP/iBINP compared with that obtained with the other treatments further supported the favorable T cell response (Figure 7B). Of note, iBINP treatment reduced the frequency of intratumoral Tregs (Figure 7C and Supplemental Figure 7D), resulting in a dramatically elevated CD8^+^/Treg ratio that favored a productive antitumor immune environment (Figure 7D). To evaluate functional T cells, we analyzed several key biomarkers on tumor-infiltrating CD8^+^ T cells. Both STING-NP and iBINP regimens enhanced the expression of the cytotoxic effector GZMB, cytokines such as IFN-γ, and proliferation marker Ki67, supporting the potent T cell activation (Figure 7, E–G, and Supplemental Figure 7E). The production of specific proinflammatory cytokines such as IFN-β, IFN-γ, IL-6, and TNF-α in tumors was then assessed using ELISA (Figure 7H). Both STING-NP and iBINP promoted the secretion of these cytokines in the tumor sites for up to 24 hours after administration (Figure 7I). By contrast, the administration of FDC and IDO1-NP monotherapy failed to trigger the release of these cytokines over and above that observed in the saline treatment group, further substantiating the synergistic effects and superior efficacy of the codelivery system. To further assess the potential risk of serious side effects such as cytokine storm, ELISA was conducted to measure the levels of various cytokines in mouse serum. The animals treated with STING-NP and iBINP exhibited elevated levels of circulating cytokines 8 hours after treatment; the levels, however, declined spontaneously after 24 hours of administration (Figure 7J), supporting the high tolerability of iBINP for therapeutic applications.

Subsequent analysis revealed upregulation of the costimulatory markers CD80/CD86 and MHC-II on DCs collected from LNs following iBINP administration (Supplemental Figure 7, F and G). Furthermore, STING therapy was observed to increase the proportion of the cytolytic CD8^+^ T cells in the spleen (Supplemental Figure 7H). The population of Tregs cells was therefore analyzed. As expected, STING-NP treatment increased the proportion of Tregs cells in spleen, whereas the combined treatment with iBINP attenuated the proportion of Tregs (Supplemental Figure 7I).

### Therapeutic efficacy of iBINP across multiple challenging cancer models.

**Figure 8:**
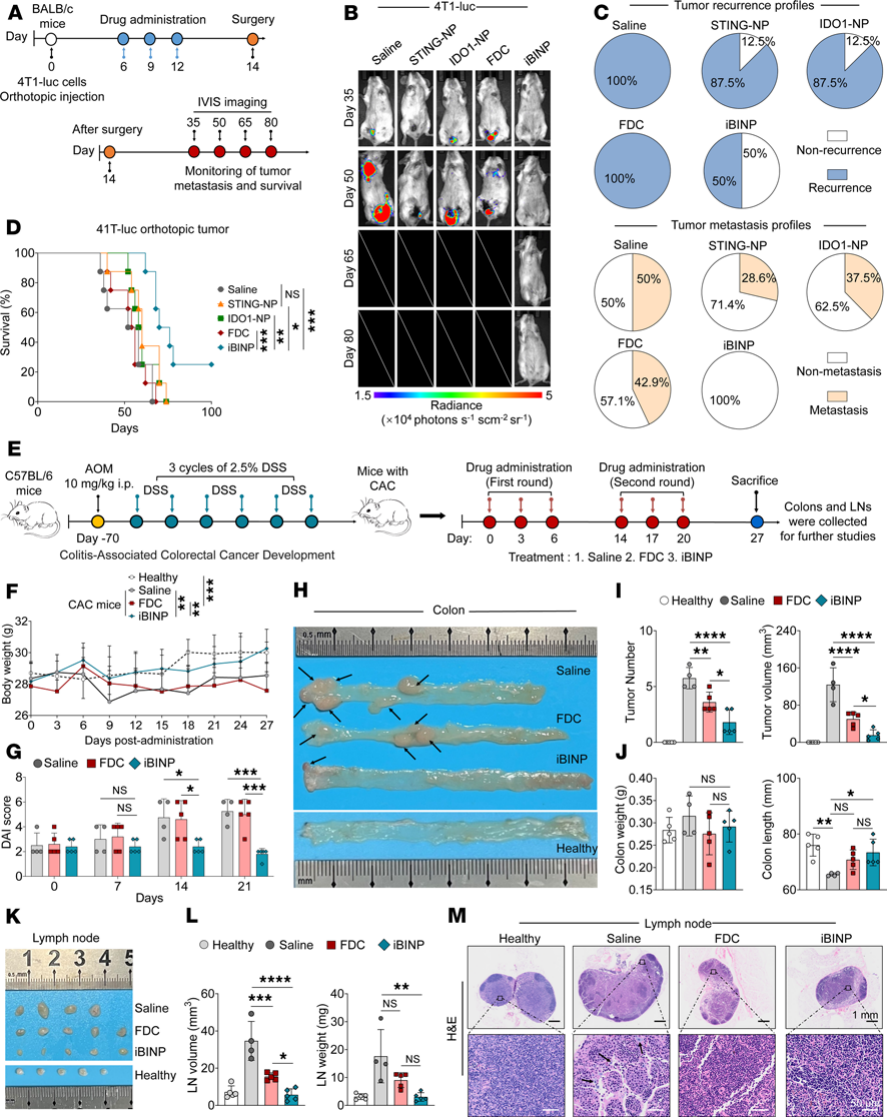
iBINP demonstrates robust therapeutic efficacy in both metastatic breast cancer and primary colorectal cancer models. (**A**) Schematic of the experimental design. Primary 4T1-tumors were surgically resected on day 14 after 3 doses of treatment, followed by long-term monitoring for metastasis. (**B** and **C**) In vivo bioluminescence imaging of tumor metastasis. Representative images of mice at the indicated time points (**B**) and analysis of tumor recurrence and metastasis profiles (**C**). (**D**) Survival analysis of mice from each treatment group (*n* = 8 mice/group). (**E**) Schematic illustration of immunotherapy in the CAC tumor model. Mice were treated with saline, FDC, or iBINP thrice a week for a period of 2 weeks, with a 1-week break in between. Colon and mLNs were collected for further analysis on day 27 (*n* = 4 for healthy; *n* = 5 for other groups). (**F**) Body weight of CAC mice of the various treatment groups after administration of the drugs. (**G**) DAI score of mice from the different treatment groups. (**H**) Representative images of colons from mice of the various treatment groups, with primary intestinal tumors indicated by black arrows. Various parameters such as (**I**) tumor numbers and total tumor volume as well as (**J**) size of the colon tumors and colon length demonstrated tumor progression within mice of the different groups. (**K**–**M**) Analysis of mLNs. Representative images of excised mLNs (**K**), quantification of LN volume and weight (**L**), and representative H&E-stained sections of mLNs (**M**). Scale bar: 1 mm (top); 50 μm (bottom). Data are presented as the mean ± SD. Statistical significance was determined by 1-way (**G**, **I**, **J**, and **L**) or 2-way ANOVA (**F**) for comparisons, and the log-rank test was used for survival analysis (**D**). NS, not significant, *P < 0.05, **P < 0.01, ***P < 0.001 and ****P < 0.0001.

Having obtained favorable outcomes in naive and STING-resistant tumor models, the efficacy of the iBINP therapy was further evaluated in other models of cancer. Cancer metastasis is the leading cause of death globally (31). We thus assessed its ability to prevent postsurgical metastasis in an orthotopic 4T1-luc breast cancer model. Following treatments, primary tumors were surgically resected, and mice were monitored for metastatic relapse by in vivo imaging system (IVIS) (Figure 8A). The IVIS imaging results showed that iBINP had antimetastatic effect, whereas control groups developed extensive metastatic disease (Figure 8B). This eventually contributed to a survival benefit, with half of iBINP-treated mice being tumor-free and 25% surviving the entire study period (Figure 8, C and D). These results were consistent with H&E staining of lungs and TDLNs; a remarkable reduction in metastatic foci in the iBINP-treated mice was observed (Supplemental Figure 8, A and B).

Finally, we extended the efficacy testing to a clinically relevant colorectal cancer model in mice that were induced by chronic inflammatory conditions (Figure 8E). This colitis-associated colorectal cancer (CAC) model was established in C57BL/6J mice by intraperitoneally injecting azoxymethane (AOM) followed by 3 cycles of free drinking of dextran sodium sulfate (DSS) (32). The iBINP therapy was found to be well tolerated in the CAC mouse model, as indicated by the absence of weight loss compared with that of the healthy controls (Figure 8F). Disease activity index (DAI) scores were employed for evaluating the health status of the CAC mouse model subjected to various treatments, which revealed that symptoms such as fecal occult blood, diarrhea, and weight loss were alleviated with iBINP treatment over time (Figure 8G). Additionally, the sizes and numbers of tumors were found to be notably suppressed upon iBINP treatment (Figure 8, H and I, and Supplemental Figure 8D). Colon length, a crucial indicator of the progression of CRC, was found to vary with different treatments due to differences in the tumor burden. Remarkably, iBINP-treated mice showed normal colon length, which was akin to that of healthy controls (Figure 8J). H&E and Ki67 staining corroborated the antitumor efficacy of the treatments, with the tumor sections from iBINP-treated mice displaying fewer colonic nodules and lower expression of Ki67 (Supplemental Figure 8, D and E). Furthermore, the expression of IDO1 was also elevated in tumors from the iBINP-treated mice owing to potent STING activation (Supplemental Figure 8F). To further elucidate the effect of different treatments on antitumor immunity, the colons from the mice were analyzed for CD8a and FOXP3 expression using IHC (Supplemental Figure 8, G and H). Differential changes were also observed in the size and weight of mesenteric lymph node (mLNs). Consistent with the observations of tumor growth, the mLNs from saline-treated mice were larger than those from the other groups (Figure 8, K and L). H&E staining of the mLNs confirmed the absence of metastatic burden but revealed the presence of inflammatory cells in the saline-treated mice (Figure 8M). Collectively, these results provide compelling evidence that STING activation and IDO restriction conferred by iBINP treatment effectively control the tumor growth and metastatic burden in two distinct and highly challenging preclinical models.

## Discussion

The immune-desert TME is devoid of cytotoxic CD8^+^ T lymphocytes, NK cells, and APCs, which necessitates the development of therapeutic strategies for overcoming resistance to immune checkpoint blockade therapy and prime T cell–based antitumor immunity (21, 24, 33, 34). The STING pathway triggers innate immune responses to cytosolic double-stranded DNA (dsDNA) in cancer cells and is a major determinant of T cell infiltration (9, 35). The tumor-derived cytosolic dsDNA binds cGAS, which produces the second messenger cGAMP that subsequently binds STING to stimulate the type I IFN–driven inflammatory response, including the production of T cell chemokines (36–38). However, these CDNs exhibit poor drug-like characteristics, limiting their clinical applicability. Moreover, the clinical outcomes associated with the intratumoral administration of STING agonists are underwhelming. These issues have been addressed through the development of several synthetic nonnucleotide STING agonists (8, 39, 40). However, the disparate roles of pharmacological STING activation complicate the therapeutic outcomes associated with anticancer therapy that relies on STING agonists. Treatment with STING agonists can induce antitumor responses via the increased secretion of IFNs and lymphocyte infiltration, which contributes to tumor control. An enhancement in productive T cell priming via cDC1 occurs under conditions of STING activation (9). Conversely, STING downregulation may contribute to the development of resistance to immune effectors in various models of cancer (41). Several research groups, including ours, have demonstrated that stimulation of the STING pathway elicits a robust immune response, which accompanies an increase in the expression of canonical immune-stimulatory genes. These results compellingly confirm the antitumorigenic role of STING/IFN signaling. However, several other studies have shown that sustained exposure of TME to STING stimulation potentially leads to adverse effects such as cytokine storms and the death of immune cells (42). Moreover, protumorigenic effects of the cGAS/STING pathway were observed in some cancer models. STING activation results in tumor outgrowth and therapeutic resistance (18, 19). We indeed verified that treatment with STING agonists such as MSA-2 induced IDO1 overexpression in cells of the tumor as well as those of the immune system. Activation of the IDO pathway leads to the depletion of Trp and undesired changes in downstream factors that inhibit effector T cells and promote the proliferation and differentiation of Tregs, ultimately favoring an immune-suppressive TME.

To address the abovementioned contradiction and overcome STING–induced immunoregulatory mechanisms, we applied a noncovalent chemical strategy to generate the immunomodulatory iBINP that facilitate the activation of the innate immune response while simultaneously restricting immune escape. This platform was constructed from the MSA-2 (STING agonist) and NLG919 (IDO1 inhibitor) prodrugs. MSA-2, a recently identified small-molecule nonnucleotide STING agonist, holds great promise as an immuno-oncology agent; however, its clinical application has been impeded by delivery obstacles, including low oral bioavailability, limited cellular uptake, and systemic inflammatory toxicity (8). For example, we found that intravenous administration of free MSA-2 caused body weight loss, indicating considerable toxicity. To expand the repertoire of these immunomodulatory agents, this potent STING agonist was utilized for the development of a self-deliverable and self-activated nanoplatform amenable to systemic administration. Our “PUFAylation” approach, exploiting a polyunsaturated fatty acid (e.g., DHA) for chemical drug derivatization, not only addresses delivery-associated challenges, but also confers an intrinsic capacity with the tropism to immune cells. Our data indeed have shown that this binary pharmacology platform leads to preferential uptake of nanotherapeutics by desired immune cells (Figure 5). Ultimately, enhancement in tumoral and lymphatic delivery, accompanied by simultaneous partitioning to the key immune cells, far surpasses those achievable with simple coadministration of free drugs.

The IDO pathway plays an important role in immune tolerance and immune evasion by tumor cells (43). IDO1, an important indicator of T cell regulation, is closely associated with the clinical responses to immunotherapy (44). However, the clinical development of IDO1 inhibitors, including NLG919 used in this study, has been largely unsuccessful (45). The possible reasons could be considered as delivery obstacles such as suboptimal pharmacokinetics, low tumor penetration, and/or the heterogenicity in IDO1 expression across different cancer types and individual patients. Interestingly, in our study, we observed the relapse of subcutaneous MC38 tumors after 3 doses of STING monotherapy, while iBINP treatment exhibited enhanced in vivo efficacy in driving tumor regression and inducing durable, tumor-specific immune memory. Furthermore, in vivo studies using the MSA-2–resistant tumor model demonstrated the superior antitumor efficacy of iBINP compared with that of monotherapies with each component drug. These findings imply that addressing an immunosuppressive TME, such as inhibition of the IDO1 escape mechanism, can lead to boosted antitumor immunity. To further provide unequivocal evidence for this hypothesis, efficacy studies using Ido1-expressing and Ido1-knockout 4T1 breast cancer models showed that the IDO1-NP potently suppressed tumor growth with high Ido1 expression. Conversely, in tumors lacking expression of IDO1, IDO1-NP monotherapy failed to show similar activity, confirming that the superiority of the iBINP platform is dependent on inhibitory rewiring of the IDO1 pathway.

In summary, our findings reveal an immunoregulatory circuit in cancer, with STING monotherapy contributing to IDO1-induced immune evasion via the IFN-γ–IDO axis, which subsequently subdues T cell–mediated antitumor responses. To relieve this negative circuit, a noncovalent binary chemical strategy has been described herein, which addresses the immunosuppressive mechanism. STING activation promotes the secretion of IFN-related cytokines, which further reinforces the DC-driven cross-priming of the antitumor CD8^+^ T cell–mediated immunity and leads to tumor regression. A simultaneous inhibition of IDO1 was incorporated herein to prevent immune escape attributable to STING-induced regulatory mechanisms. Consequently, this synergistic iBINP system increased the proportion of intratumoral CD8^+^ T cells while decreasing the infiltration of Tregs. Collectively, this work highlights that simultaneously targeting multiple vulnerabilities via rational chemical approaches may offer considerable opportunities for achieving synergistic immune responses to elevate the efficacy and success rate of immunotherapy in cancer.

## Methods

### Sex as a biological variable.

Both male and female C57BL/6 mice were used for the MC38 and B16-OVA tumor models. Only female BALB/c mice were used for the 4T1 tumor models (both orthotopic and subcutaneous). In the models where both sexes were included, no sex-specific differences in therapeutic outcomes were observed. Therefore, sex was not considered as a biological variable in this study.

### Compounds.

DHA was purchased from MedChemExpress (HY-B2167). DTT (A100281) was purchased from Sangon Biotech. Porcine liver esterase (PLE, 46058) was obtained from Sigma Aldrich. 1,2-Distearoyl-sn-glycero-3-phosphoethanolamine-N- [methoxy (polyethylene glycol) 2000] (DSPE-PEG2000, F01008) was purchased from A.V.T. Pharmaceutical Co. Ltd. All other and solvents were purchased from J&K Chemical or TCI Development Co. Ltd.

### Synthesis of prodrugs based on MSA-2 and NLG919.

The chemical structures of the synthesized MSA-2 and NLG919 prodrugs were characterized using proton nuclear magnetic resonance (¹H NMR) spectroscopy. Synthesis schemes are shown in Supplemental Figure 9. Detailed synthetic procedures are included in Supplemental Methods.

### Cell lines and culture.

The murine colon adenocarcinoma cell line MC38, murine breast cancer cell line 4T1, HEK293T cells, and human colon adenocarcinoma cell line LoVo were purchased from the Chinese Academy of Sciences Cell Bank. The mouse melanoma cell lines B16-OVA and 4T1-luc cell line were purchased from Bowers Type Culture Collection. Cells were cultured in RPMI-1640 (Biological Industries) or DMEM (Biological Industries) supplemented with 10% FBS (Vazyme, F101) and 1% penicillin-streptomycin (BDBIO, A200-100) at 37°C in a humidified atmosphere with 5% carbon dioxide. Bone marrow–derived DCs (BMDCs) were obtained from the bone marrow of C57BL/6 mice and human peripheral blood mononuclear cells (hPBMCs) were cultured in RPMI-1640 medium supplemented with 10% heat-inactivated FBS, 1% penicillin-streptomycin, 5 ng/mL IL-4 (Abclonal, RP01161), and 20 ng/mL murine GM-CSF (Abclonal, RP01206). The culture medium was partially replaced on days 3 or 4, and nonadherent cells were collected for further experiments on days 6–8.

### Tumor models.

The MC38 tumor model used for the evaluation of the antitumor efficacy of the treatment was obtained via the subcutaneous injection of MC38 cells (1 × 10^6^ cells) into the right flank of C57BL/6 mice aged 6–8 weeks. The tumor was then allowed to grow for 7 days, and after a tumor size of approximately 100 mm^3^ was attained, the mice were treated with various combinations of drugs. The treatments were administered via the tail vein on days 0, 3, and 6, while free MSA-2 was administered orally or via tail vein injection. Tumor growth and body weight were monitored and recorded every 2–3 days. Tumor volume (*V*) was calculated using the following formula: *V* = (length × width^2^)/2.

The MSA-2–resistant MC38 tumor model was obtained by conducting 3 cycles of selection for the resistant tumors. Specifically, the C57BL/6 mice were subcutaneously injected with the tumor cell line MC38. When the tumors attained volumes of approximately 100 mm^3^, STING-NP (equivalent to a 30 mg/kg dose of MSA-2) was administered thrice via tail vein injection. The tumor growth was then monitored, and nonresponding (NR) mice (defined as those exhibiting continuous tumor growth, with no effect of the drug) were selected. The tumors of NR mice were excised, cut into uniform small pieces of approximately 10 mm^3^ volume, and subcutaneously transplanted into a new batch of C57BL/6 mice. Three such cycles were carried out to establish an MSA-2–resistant MC38 tumor model. Tumor volume and survival were monitored as previously described.

The AOM/DSS–induced CAC model was established as follows: WT C57BL/6 mice were intraperitoneally administered a single dose (10 mg/kg) of AOM (MP Biomedicals, 218397125). Seven days following the AOM administration, 2.5% DSS (MP Biomedicals, 9011-18-1) (w/v) was added to the drinking water for a duration of 1 week. Primary tumors were found to develop after 3 such cycles of intake of 2.5% DSS via drinking water. The CAC mice were then administered FDC or the iBINP at a dosage equivalent to 30 mg/kg MSA-2/10 mg/kg NLG919. Body weights of the mice were recorded every 3 days from the initiation of treatment. The mice were euthanized at the end of the study, and the colon was collected for analyzing the numbers and sizes of the tumors as well as colon weight and length. The tissues were fixed with 4% paraformaldehyde and subjected to H&E as well as immunofluorescence staining.

For the orthotopic breast cancer model, female BALB/c mice (8 weeks old) were anesthetized, and 4T1-luc cells (1 × 10^5^) expressing luciferase were injected into the fourth mammary fat pad. Tumor growth was monitored by palpation. Seven days after inoculation, mice were randomly assigned to treatment groups and received intravenous injections of Saline, STING-NP, IDO1-NP, FDC, or iBINP on days 7, 10, and 13 post-inoculations. On day 14, the primary tumors were surgically resected from all mice under anesthesia. Post-surgical monitoring for metastatic relapse and survival was conducted and performing bioluminescence imaging using an IVIS (Biolight Biotechnology Co. Ltd.) on days 35, 50, 65, and 80 after tumor cell injection.

### IDO1 enzyme activity assay.

MC38 cells were plated at a density of 4 × 10^4^ cells per well in RPMI-1640 media (phenol red-free) supplemented with 80 μM L-tryptophan (L-Trp, TCI, T0541). To induce IDO1 expression, 50 ng/ml of mouse recombinant IFN-γ (Abclonal, RP01070) was added to each well and incubated for 24 hours. Subsequently, free NLG919 or IDO1-NP (NLG919, 10 μM) was introduced into the cell culture, followed by further incubation for 24 hours at 37°C. The culture supernatant (200 μL) was collected from each well for further analysis, as described previously (46). Briefly, the supernatant from each well was mixed with 10 μL of 30% trichloroacetic acid (TCI, T0369) to precipitate proteins. Following centrifugation, the concentrations of Trp and L-Kynurenine (L-Kyn, TCI, K0016) in the cleared supernatant were quantified by high-performance liquid chromatography (HPLC). The levels of Trp and Kyn were determined by monitoring absorbance at 280 nm and 360 nm, respectively.

### scRNA-seq and analysis.

Cells derived from MC38 xenograft tumors on day 7 after treated with MSA-2 (intratumoral) were sorted for scRNA-seq. scRNA-seq was performed using the 10X Genomics Chromium Next GEM Single Cell 3′ Reagent Kits by Shanghai OE Biotech Co. Ltd. Subsequently, samples were processed following the manufacturer’s protocol and sequenced on an Illumina NovaSeq sequencer. The Cell Ranger analysis pipeline (v7.1.0) was employed to generate gene count matrices for each cell per sample. These matrices were imported into Seurat (v5) for integration and subsequent analyses. After quality filtering, a total of 29,003 high-quality immune cells (14,386 from saline-treated tumors and 14,617 from MSA-2–treated tumors), with a median expression of 2,858 genes, were retained for further analysis. To visualize clustering results, nonlinear dimensional reduction was performed using the t-distributed stochastic neighbor embedding (t-SNE) method. Cluster biomarkers were identified using the FindAllMarkers function. Clusters were defined based on highly expressed genes specific to each cluster. DEGs were selected using the FindMarkers function (test.use = presto) in Seurat. *P* < 0.05 and |log_2_foldchange| > 0.58 was set as the threshold for significantly differential expression. Gene Ontology enrichment and KEGG pathway enrichment analysis of DEGs were performed using R based on the hypergeometric distribution.

### Statistics.

Statistical analysis was conducted using Prism 9.0 (GraphPad) and R (v4.1.3) software. The results are expressed as mean ± SD as specified. The number of independent experiments or replicates and details of the tests employed for statistical analysis, including 2-tailed unpaired Student’s *t* test as well as 1-way or 2-way ANOVA, are provided in the figure legends. Survival curves were generated using the Kaplan-Meier method, and *P* values between groups were calculated using the log-rank test. A *P* value less than 0.05 was considered significant.

### Study approval.

All animal studies were conducted in accordance with institutional guidelines for the care and use of laboratory animals. All protocols for animal experiments were approved by the Ethics Committee of the First Affiliated Hospital, Zhejiang University School of Medicine. The use of deidentified human specimens in this study was approved by the Ethics Committee of the First Affiliated Hospital, Zhejiang University School of Medicine (approval no. [2025B] IIT-0986). The requirement for written informed consent was waived by the ethics committee because the research involved no direct contact with participants and posed no risks to privacy or commercial interests.

### Data availability.

Values for all individual data points are reported in the Supporting Data Values file. All unedited blots are also included in the raw Western blot document. The scRNA-seq data reported in this paper have been deposited in NCBI’s Gene Expression Omnibus (GEO GSE306421). All other underlying data are available upon request.

## Author contributions

HW and FM designed the study. FM, HZ, SW, and BL conducted experiments. FM, BL, and XC analyzed the data. FM and HZ wrote the manuscript. HW and FM reviewed and revised the manuscript. HW supervised the study.

## Funding support

- “Pioneer” and “Leading Goose” R&D Program of Zhejiang Province grants (2025C01217).
- Natural Science Foundation of Shandong Province (ZR2023ZD59).
- National Natural Science Foundation of China (82273490 and 82073296).
- Nonprofit Central Research Institute Fund of Chinese Academy of Medical Sciences (2023-PT320-02).
- Research Project of Jinan Microecological Biomedicine Shandong Laboratory (JNL-2025007B).

## Supplementary Material

Supplemental data

Unedited blot and gel images

Supporting data values

## Version 1 10/23/2025

In-Press Preview

## Version 2 12/15/2025

Electronic publication
