# Supplementary material for: Targeting STING-induced immune evasion with nanoparticulate binary pharmacology improves tumor control in mice
Source: J Clin Invest. 2025 Oct 23;135(24):e192397. doi: 10.1172/JCI192397 (PMC12700560; doi:10.1172/JCI192397)
Supplement: Supplemental data [file jci-135-192397-s009.pdf]

Supplemental information for

Targeting STING–induced immune evasion with nanoparticulate binary pharmacology improves tumor control in mice

Fanchao Meng,[1,2] Hengyan Zhu,[1] Shuo Wu,[1] Bohan Li,[1] Xiaona Chen,[1] and Hangxiang Wang[1,2,]*

[1] The First Affiliated Hospital, NHC Key Laboratory of Combined Multi-Organ Transplantation, Collaborative Innovation Center for Diagnosis and Treatment of Infectious Diseases, State Key Laboratory for Diagnosis and Treatment of Infectious Diseases, Zhejiang University School of Medicine, Zhejiang Province, Hangzhou, 310003, P. R. China

[2] Jinan Microecological Biomedicine Shandong Laboratory, Jinan, Shandong Province, 250117, P. R. China

***Corresponding Author:** Hangxiang Wang, The First Affiliated Hospital, Zhejiang University School of Medicine, 79 Qingchun Road, Hangzhou 310003, P. R. China. E-mail: wanghx@zju.edu.cn

This PDF file includes:

Supplemental Figures 1 to 9
Supplemental materials and methods

Supplemental figures

Supplemental Figure 1

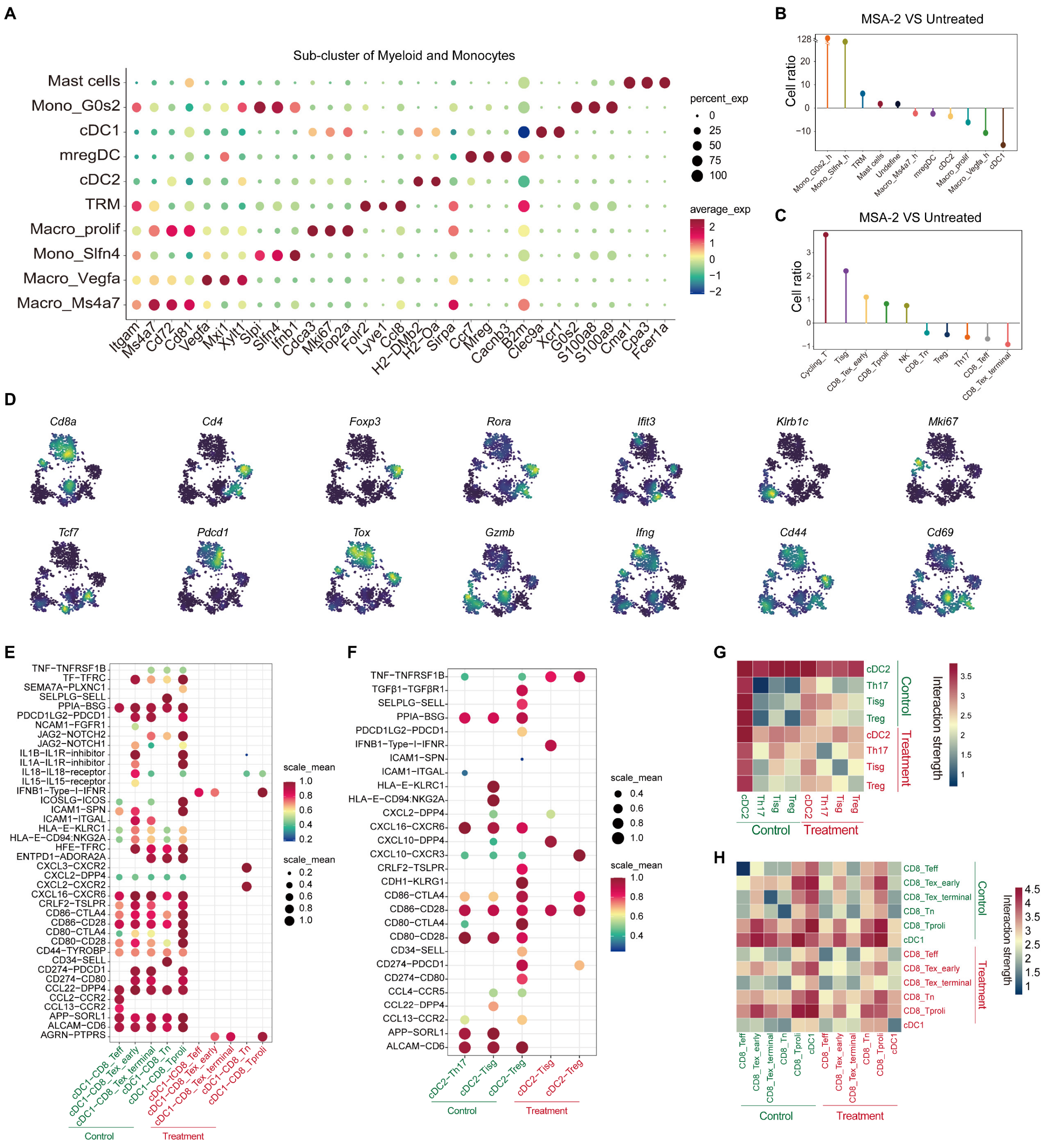

Supplemental Figure 1. **Intratumoral injection of MSA-2 elicits a potent pro-inflammatory response and causes immune cell death. related to Figure 1. (A)** Heatmap displaying the expression of canonical marker genes used to define myeloid and monocyte subclusters identified in MC38 tumors. **(B).** Cell ratio changes of different myeloid cell sub-clusters (MSA-2 versus Untreated).

(C). Cell ratio changes of different NK/T cell sub-clusters (MSA-2 versus Untreated). **(D).** t-SNE plots showing the expression of functional cell markers in different NK/T cell populations. **(E-F)** The bubble plot primarily illustrated the cell communication between cDC1 and cDC2 cells and T cells. **(G-H)** The clustering heatmap shows the extent of cell communication between DC cells and T cells within the untreated and MSA-2 intratumoral injection groups.

Supplemental Figure 2

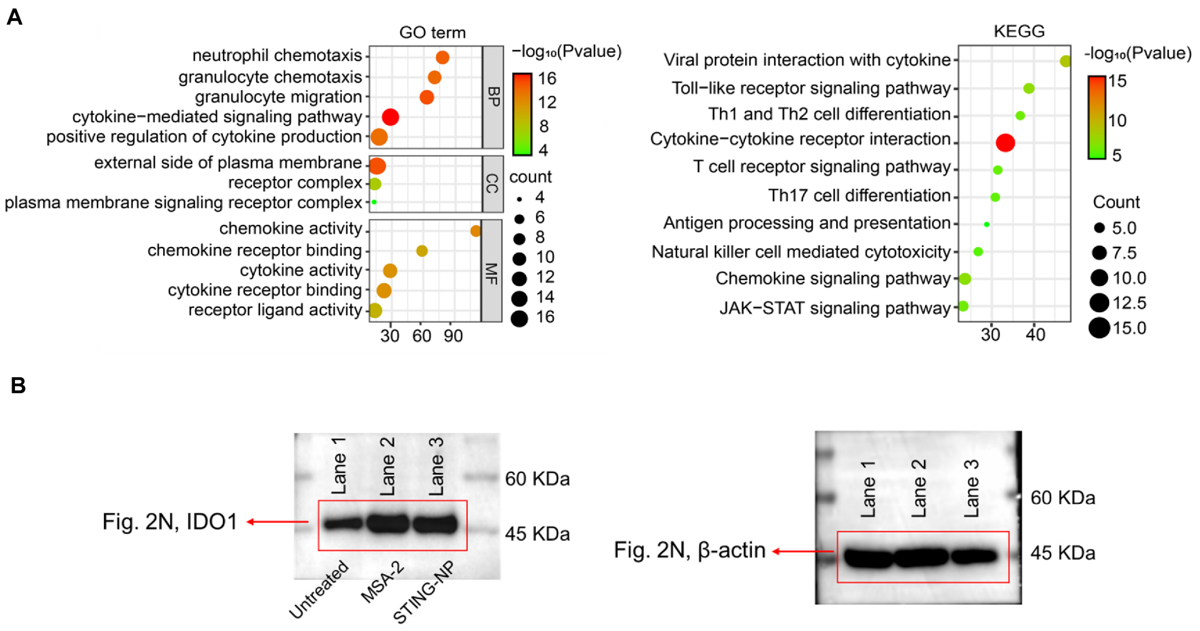

Supplemental Figure 2. **STING pathway activation upregulates the expression of *Ido1.* related to Figure 2.** **(A)** Analysis of GO and KEGG terms of the overlapping DEGs from GEO datasets (GSE134129, GSE159825, and GSE204825). The input for GO/KEGG Enrichment Analysis were based on *P* Value. Significance *P* was determined using the DESeq2 corrected for multiple comparisons. **(B)** Full and uncropped blot related to Figure 2N. The blot shows the full image for the western blot presented in Figure 2N. The lane 3, corresponding to the STING-NP treatment group, was not included in the main figure to maintain narrative consistency with the main text, as the STING-NP group is introduced later in the manuscript.

Supplemental Figure 3

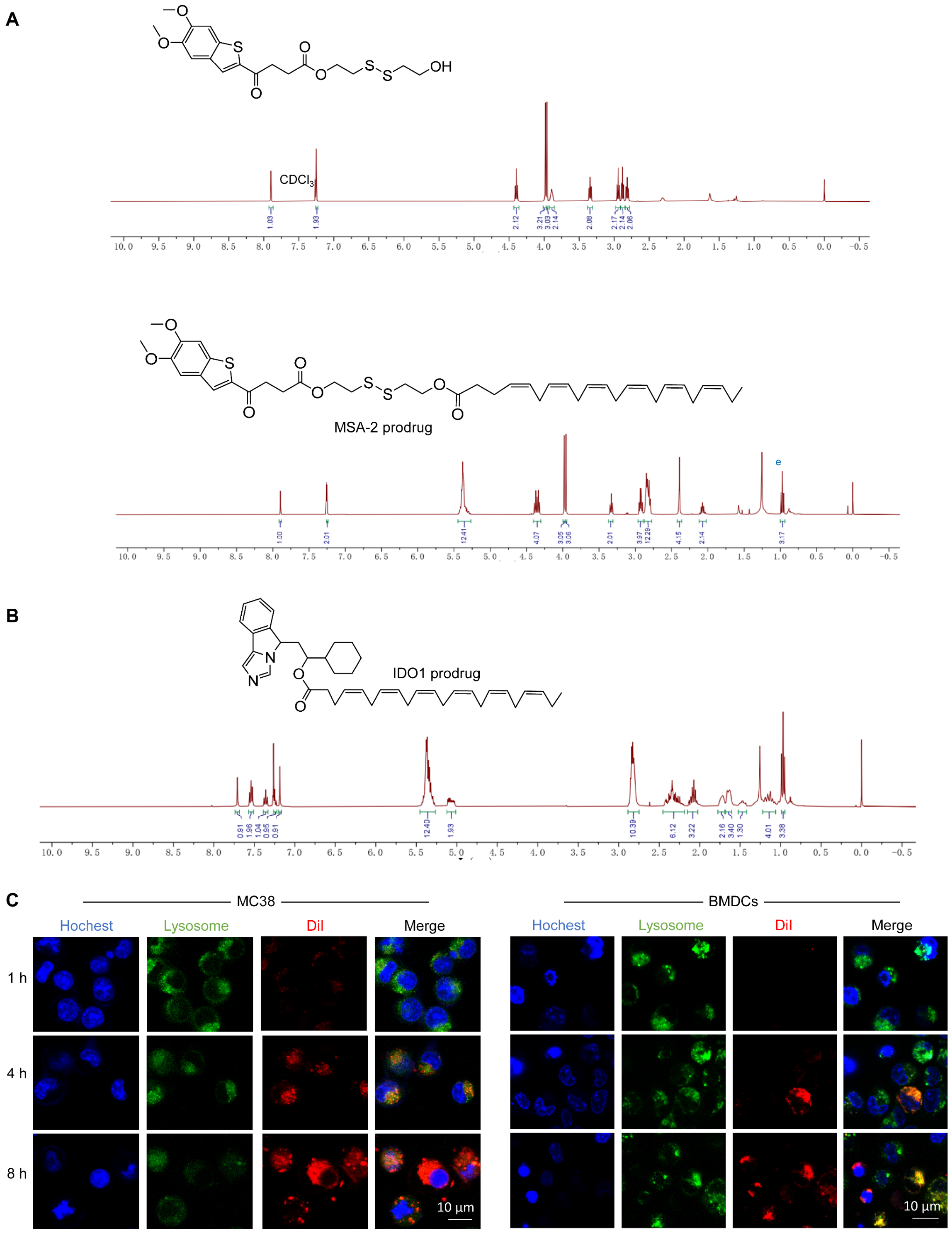

Supplemental Figure 3. **Preparation and characterization of the iBINP, related to Figure 3. (A, B)** ¹H NMR spectra of MSA-2-SS-DHA (prodrug **1**) and NLG919-DHA (prodrug **2**), confirming the successful conjugation of the respective parent drugs to the docosahexaenoic acid (DHA) moiety. **(C)**

Confocal laser scanning microscopy images showing the time-dependent cellular uptake and intracellular trafficking of DiI-labeled iBINP (red) in MC38 tumor cells and bone marrow-derived dendritic cells (BMDCs). Cell nuclei were stained with Hoechst 33342 (blue), and lysosomes were stained with LysoTracker Green (green). Co-localization of DiI with LysoTracker at early time points (1 h, 4 h) followed by separation at a later time point (8 h) indicates efficient endo-lysosomal escape of the nanoparticles. Scale bar, 10 μm.

Supplemental Figure 4

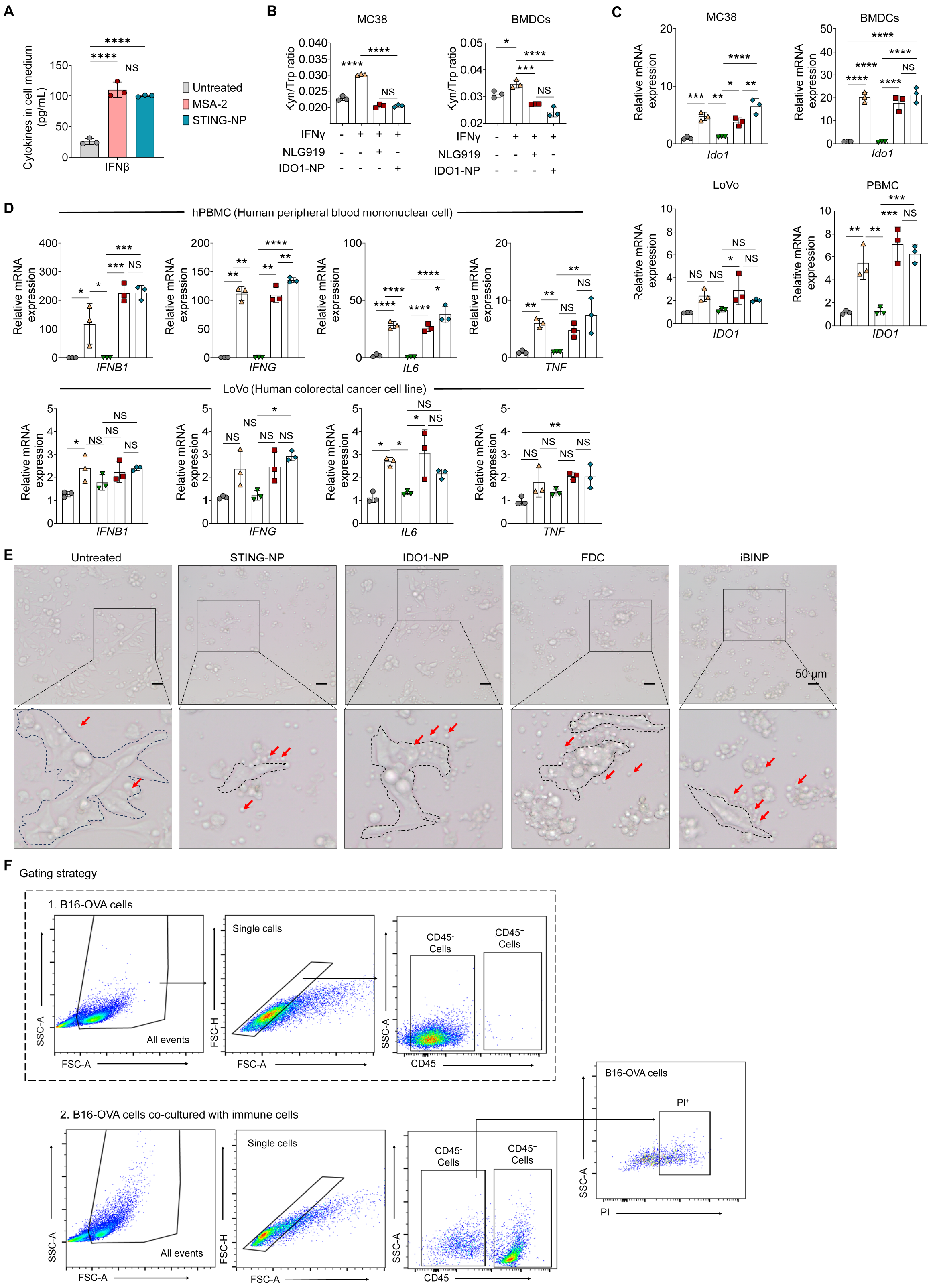

***Supplemental Figure** 4. **Combination therapy with STING agonist and IDO1 inhibitor improves antitumor immunotherapy under *in vitro* conditions, related to figure 4. (A)** ELISA quantification

of IFNβ secreted by bone marrow-derived dendritic cells (BMDCs) after treatment with free MSA-2 or STING-NP (*n* = 3). **(B)** Kynurenine/Tryptophan (Kyn/Trp) ratio in the supernatant of IFNγ-stimulated MC38 cells and BMDCs treated with free NLG919 or IDO1-NP (*n* = 3). **(C)** qRT-PCR analysis showing upregulation of *IDO1* mRNA in tumor or immune cells (*n* = 3). **(D)** Representative bright-field microscopy images of B16-OVA cells after 24 hours of co-culture with CTLs under different treatment conditions: Untreated, STING-NP, IDO1-NP, FDC, and iBINP. The bottom row shows magnified views of the areas indicated by the boxes in the top row. **(E)** Representative microscopy images of B16F10-OVA tumor cells co-cultured with OVA-specific cytotoxic T lymphocytes (CTLs) after various treatments. Pronounced adhesion of CTLs to tumor cells is observed in STING-NP, FDC, and iBINP-treated groups, indicative of immune recognition. Scale bar, 50 μm. **(F)** Flow cytometry gating strategy for the tumor cell killing assay shown in Fig. 4K. Live, single cells were first gated, followed by identification of the CD45-negative tumor cell population to specifically analyze their viability via propidium iodide (PI) staining. Statistical significance was determined by one-way ANOVA with Tukey's multiple comparison test. Data is depicted as the mean ± SD. NS, not significant. $P < 0.05$, ** $P < 0.01$, *** $P < 0.001$, **** $P < 0.0001$.

Supplemental Figure 5

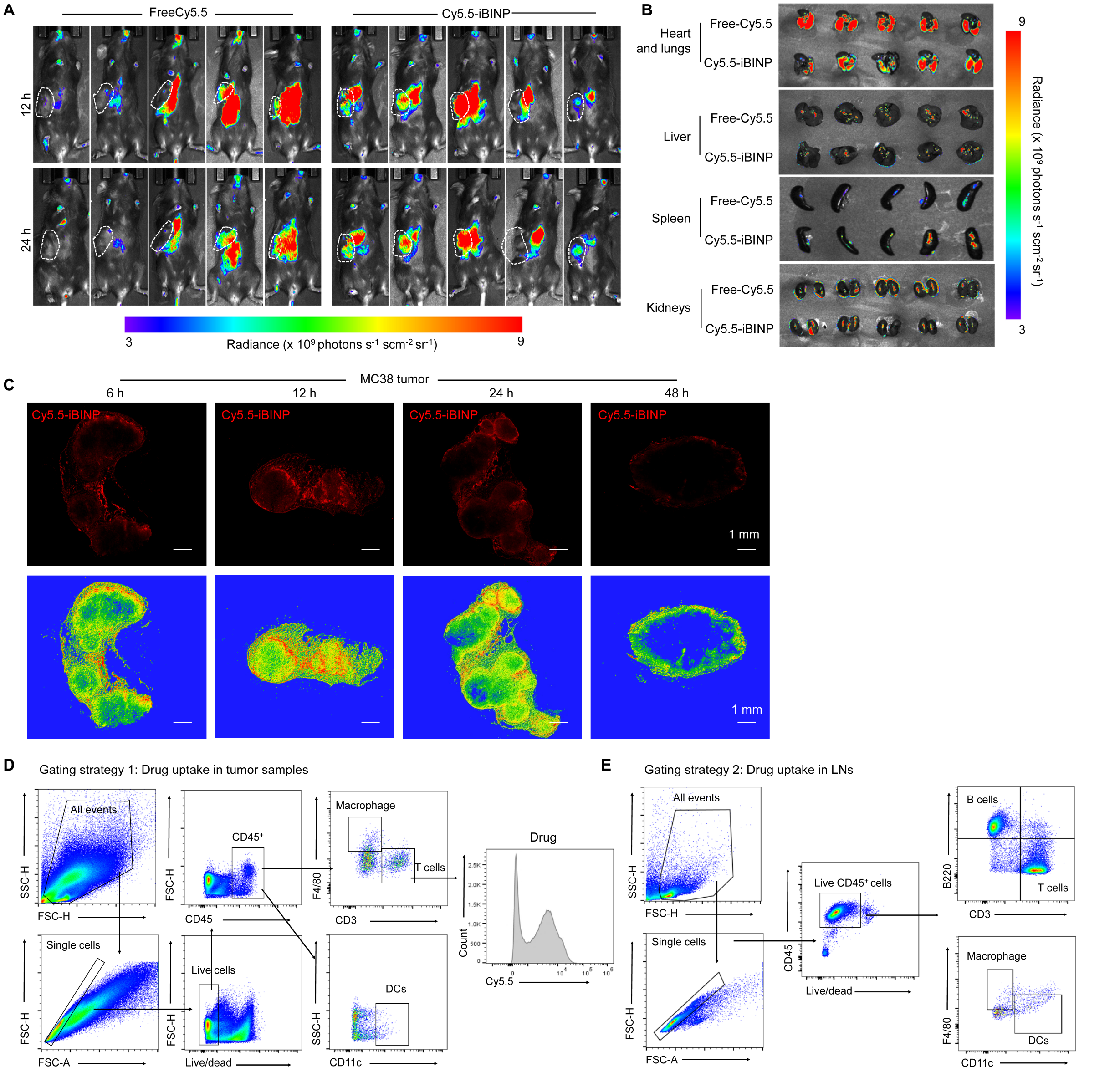

Supplemental Figure 5. **iBINP enhances tumor accumulation and lymphatic delivery, promoting immune cell targeting, related to figure 5. (A)** In vivo fluorescence imaging of MC38 tumor-bearing mice at 12 and 24 hours after intravenous injection of free Cy5.5 or Cy5.5-labelled nanoplatform (Cy5.5-iBINP). Tumor regions are outlined with dashed lines (*n* = 5 mice/group). **(B)** Ex vivo fluorescence imaging of major organs (heart, lungs, liver, spleen, kidneys) harvested from mice 24 hours post-injection, showing the biodistribution of free Cy5.5 versus the nanoplatform (*n* = 5 mice/group). **(C)** Time-course in vivo fluorescence imaging of MC38 tumors showing sustained accumulation of Cy5.5-iBINP over 48 h. **(D, E)** Flow cytometry gating strategies used to identify major

immune cell populations and quantify nanoparticle uptake (Cy5.5$^+$ cells) in dissociated tumors **(D)** and tumor-draining lymph nodes (LNs) **(E)**. Cell populations include tumor cells (CD45$^-$), T cells (CD45$^+$CD3$^+$), macrophages (CD45$^+$F4/80$^+$), dendritic cells (CD45$^+$CD11c$^+$), and B cells (CD45$^+$B220$^+$).

Supplemental Figure 6

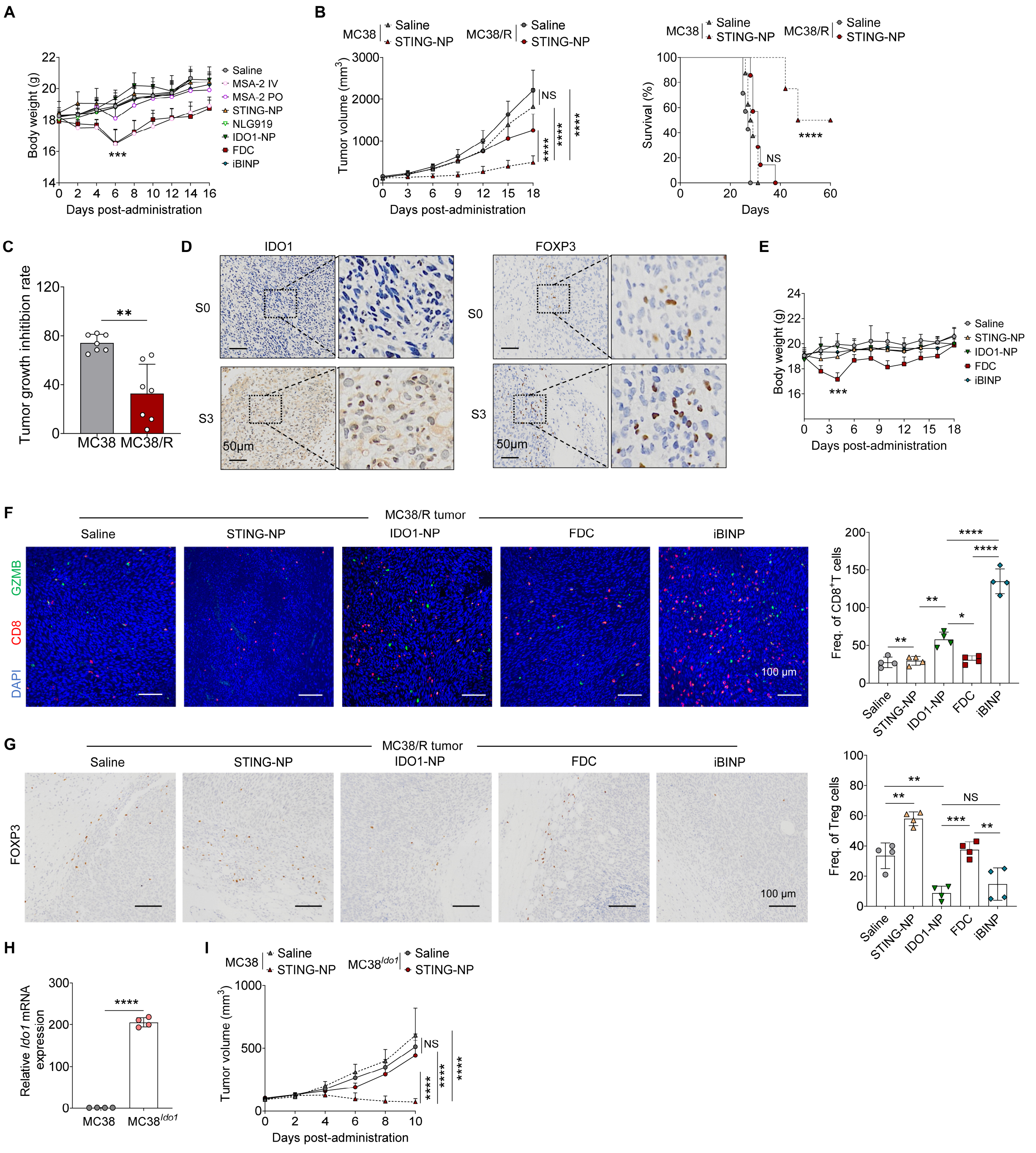

Supplemental Figure 6. **iBINP overcomes acquired resistance to STING monotherapy by targeting the IDO1 feedback loop, related to figure 6. (A)** Body weight of mice bearing MC38 tumors during treatment with various formulations, including Saline, free STING agonist (MSA-2, p.o. or i.v.), free IDO inhibitor (NLG919), single-drug nanoparticles (STING-NP, IDO1-NP), a free drug combination (FDC), and the integrated nanoplatform (iBINP) (*n* = 8 mice/group). **(B)** Tumor growth curves **(B)** and survival analysis of mice bearing MC38 tumors treated with Saline or STING-NP (*n* = 8 for parental MC38 tumors; *n* = 7 for MC38 tumors). **(C)** Tumor growth inhibition rates in parental MC38 tumors versus MC38/R tumors. **(D)** Immunohistochemistry (IHC) staining showing a progressive increase in IDO1 expression and $FOXP3^+$ Treg infiltration in successive generations (S0 to S3) of STING-agonist resistant tumors. Scale bar, 50 μm. **(E)** Body weight of mice bearing MC38/R tumors during treatment (*n* = 7 for Saline and STING-NP; *n* = 8 for other groups). **(F)** Representative immunofluorescence images and quantification of intratumoral $CD8^+$ T cells (green) and granzyme B (GZMB, red) expression in MC38/R tumors from different treatment groups. Shown are representative lower-magnification images corresponding to the higher-magnification fields presented in Figure. 6I. Nuclei were stained with DAPI (blue) (*n* = 4). Scale bar, 100 μm. **(G)** Representative immunohistochemistry images and quantification of intratumoral $FOXP3^+$ regulatory T cells (Tregs) in MC38/R tumors (*n* = 4). **(H)** Verification of the overexpressing of *Ido1 mRNA* in $MC38^{Ido1}$ cell line. **(I)** Tumor growth curves of mice bearing MC38 or $MC38^{Ido1}$ Tumors treated with Saline or STING-NP (*n* = 4 mice/group). Data are presented as mean ± SD. Statistical analysis by two-way ANOVA with Turkey's multiple comparisons test (A, B, E, G, I), log-rank test (B), one-way ANOVA with Turkey's multiple comparisons test (F, G), or Student's t test (C, H). NS, not significant, *P < 0.05, **P < 0.01 and ***P < 0.001, ****P < 0.0001.

Supplemental Figure 7

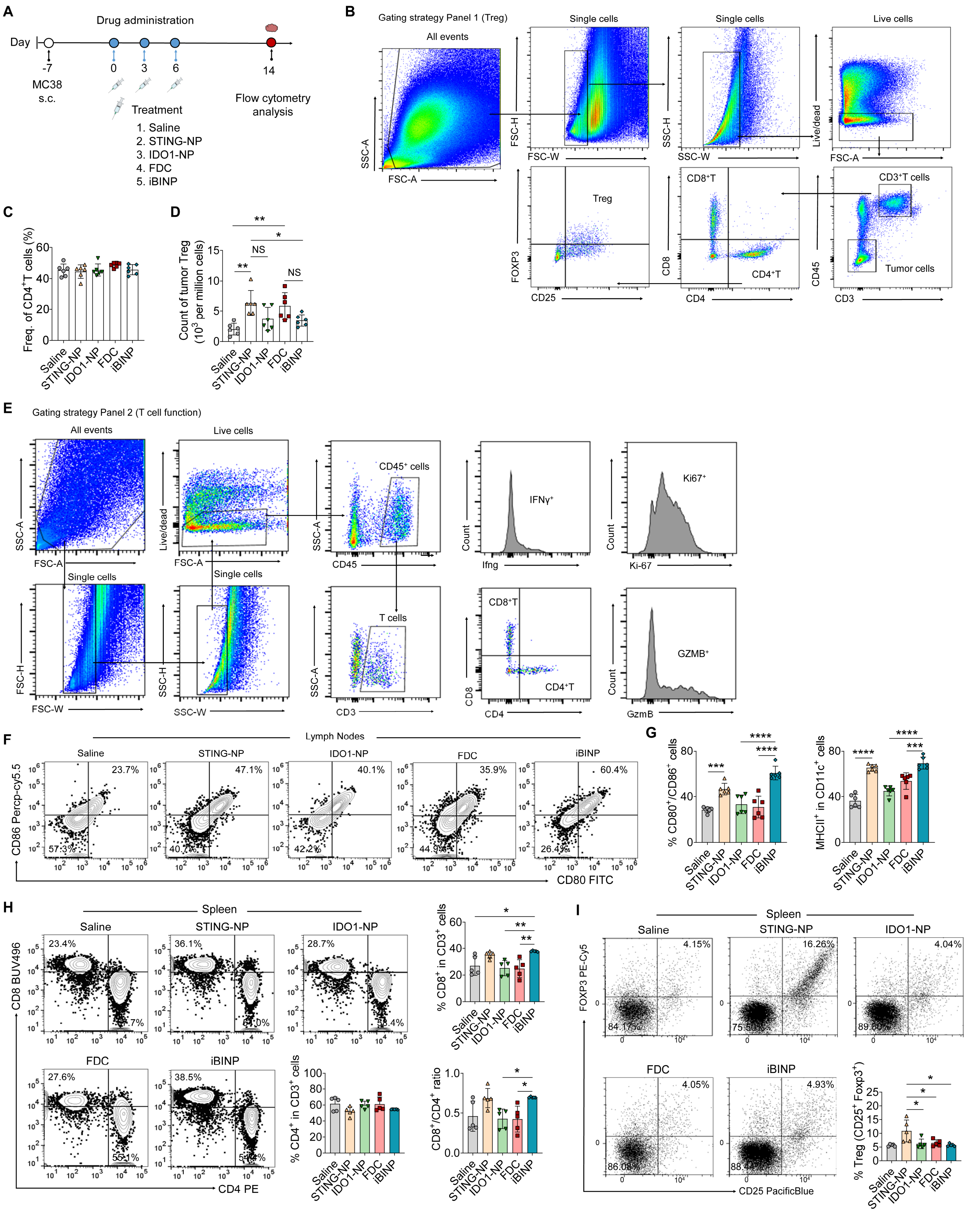

Supplemental Figure 7. **iBINP reshapes the tumor immune microenvironment by promoting a robust cytotoxic T cell response, related to figure 7. (A)** Schematic of the treatment and analysis schedule for the subcutaneous MC38 tumor model. Mice were treated with Saline, STING-NP, IDO1-NP, FDC, or iBINP. **(B)** Representative flow cytometry gating strategy for identifying intratumoral

regulatory T cells (Tregs; $CD3^+CD4^+CD25^+FOXP3^+$). Live single cells were sequentially gated to identify the target population. **(C)** Quantification of the frequency of $CD4^+$ T cells within the $CD3^+$ T cell population in MC38 tumors (*n* = 6 mice/group). **(D)** Quantification of the absolute number of Tregs per million cells within tumors from treated mice (*n* = 6 mice/group). **(E)** Representative gating strategy for analyzing T cell function. Live, single $CD45^+$ immune cells were gated for $CD3^+$ T cells, which were further subdivided into $CD4^+$ and $CD8^+$ populations for analysis of interferon-γ (IFNγ), Ki67, and granzyme B (GZMB) expression. **(F, G)** Flow cytometric analysis showing the expression of co-stimulatory markers CD80/CD86 **(F)** and MHC-II **(G)** on dendritic cells ($CD11c^+$) from tumor-draining LNs (*n* = 6 mice/group). **(H)** Representative plots show $CD4^+$ and $CD8^+$ T cell populations gated on live $CD3^+$ cells. Bar graphs show quantification of their respective frequencies and the overall $CD8^+/CD4^+$ ratio (*n* = 5 mice/group). **(I)** Analysis of splenic Tregs. Representative plots and quantification of the frequency of Tregs ($CD25^+FOXP3^+$) within the $CD4^+$ T cell gate (*n* = 5 mice/group). Data are presented as mean ±SD. Statistical significance was determined by one-way ANOVA with Tukey's multiple comparison test. NS, not significant. $P < 0.05$, ** $P < 0.01$, *** $P < 0.001$, **** $P < 0.0001$.

Supplemental Figure 8

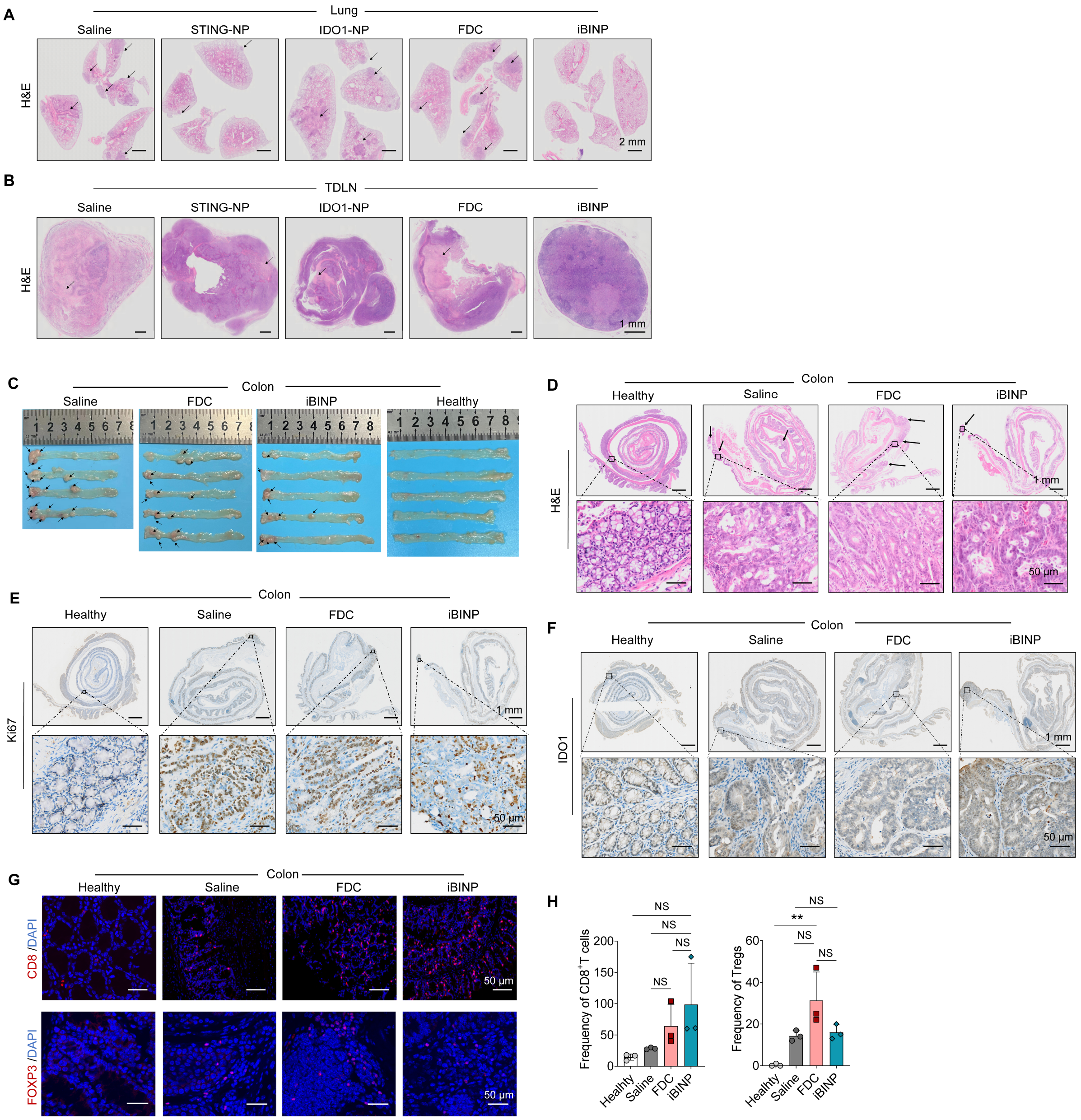

Supplemental Figure 8. **iBINP demonstrates robust therapeutic efficacy in both metastatic breast cancer and primary colorectal cancer models, related to figure 8. (A, B)** Representative H&E staining images of lung **(A)** and Tumor Draining Lymph Node (TDLN) **(B)** sections from mice in different treatment groups. Treatments include Saline, STING-NP, IDO1-NP, FDC, and iBINP. Black arrows in the lung or TDLN sections indicate metastatic foci. Scale bar: 1- or 2-mm. **(C)** Representative photographs of colons of CAC mice. Black arrows indicate the primary intestinal tumors. **(D)** Representative images of colon sections from the CAC mice stained with H&E and for evaluating tumor growth. **(E, F)** IHC analysis of colon sections from the CAC mice for Ki67 **(E)** and IDO1 **(F)** protein

indicates upregulated expression at the tumor site. **(G)** Representative immunofluorescence images following co-staining of sections of colon obtained from mice of each treatment group for CD8 and FOXP3 (red) and DAPI (blue). **(H)** Quantification of the frequency of $CD8^+$ T cells and Tregs in the colons of different groups based on immunofluorescence staining (n = 3). Data is presented as the mean ± SD. One-way ANOVA was employed for statistical analysis. NS, not significant. ** $P < 0.01$. Scale bar is 1 mm (E, F, G, top panel) and 50 μm (E, F, G bottom panel, and H).

Supplemental Figure 9

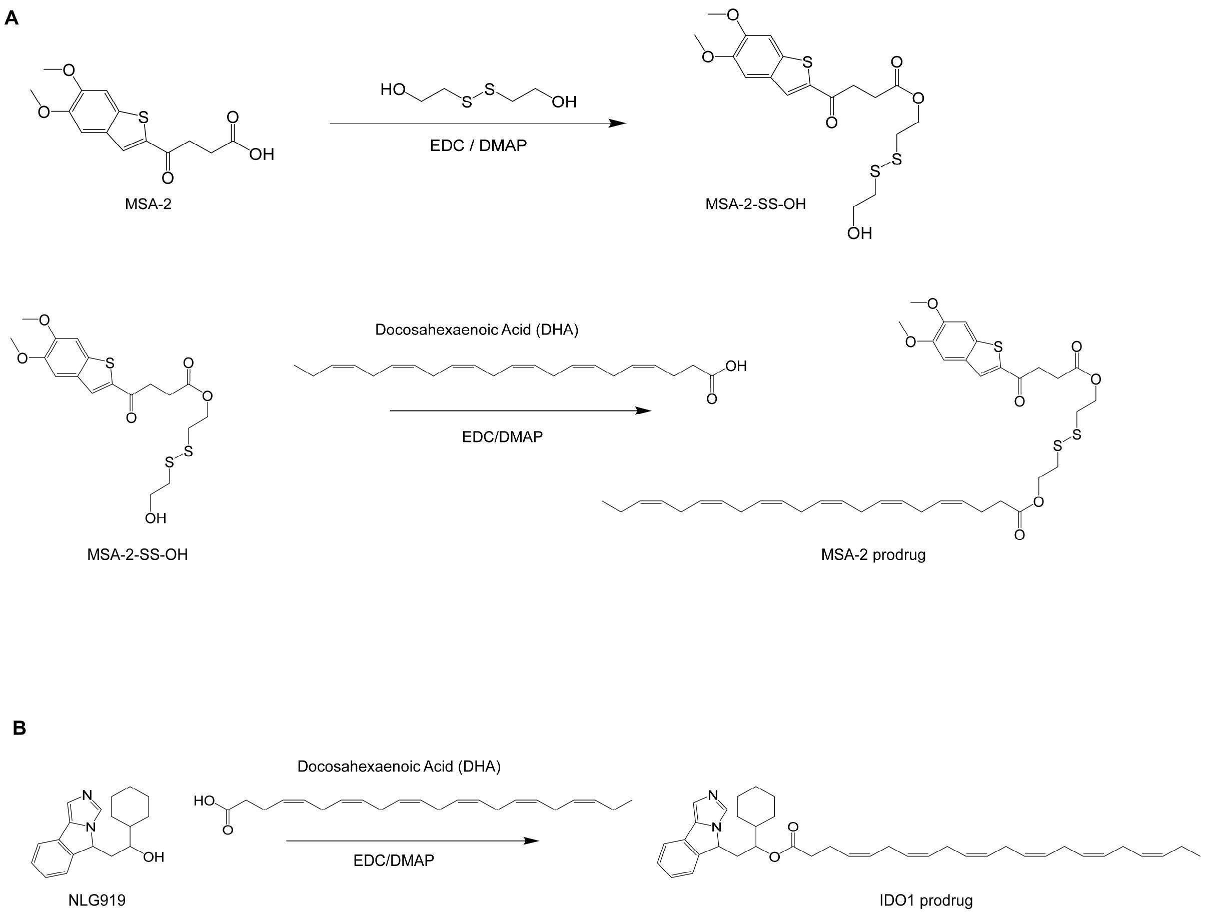

Supplemental Figure 9. Synthesis scheme of MSA-2 and IDO1 inhibitor prodrugs. **(A, B)** Two-step chemical synthesis route for the MSA-2 prodrug **(A)** and the NLG919 prodrug **(B)**. The parent drug is first conjugated with a disulfide linker (for MSA-2) or directly prepared for esterification. In the second step, the intermediate is reacted with docosahexaenoic acid (DHA) via an ester bond using EDC/DMAP as coupling agents to yield the final amphiphilic prodrug capable of self-assembly.

Supplemental materials and methods

Animals

Female BALB/c and male or female C57BL/6 mice (6-8 weeks) were sourced from the Laboratory Animal Center of Hangzhou Medical College (Hangzhou, China). The animal facility maintained a specific pathogen-free (SPF) environment with a 12/12 h light/dark cycle, a temperature of 23–26 °C, and humidity between 40–60%. In compliance with institutional animal ethics guidelines and welfare regulations, mice were humanely euthanized by CO_2 inhalation once they reached the experimental endpoints approved by institutional animal ethics committee.

Materials

BD Pharmingen™ PI Staining Buffer (550825) was purchased from BD Biosciences. Hieff Trans® Universal Transfection Reagent (40808ES03) was purchased from Yeasen Biotechnology Co., Ltd. D-Luciferin, Potassium Salt D (Yeasen, 40902ES03) was used for in vivo imaging.

Antibodies

For Western Blotting assay, anti-IRF3 rabbit mAb (D83B9, #4302), anti-phospho-IRF3 (Ser396) rabbit mAb (4D4G, #4947), anti-TBK1 rabbit mAb (D1B4, #3504), anti-phospho-TBK1 (Ser172) rabbit mAb (D52C2, #5483) and anti-β-actin mouse mAb (8H10D10, #3700) were used and purchased from Cell Signaling Technology (USA). Anti-IDO1 rabbit mAb (EPR28349-89, ab311847) were used and purchased from Abcam.

For Flow cytometry assay, APC anti-mouse CD45 (30-F11, Biolegend, 103112), BUV395 Rat Anti-Mouse CD45(30-F11, BD Biosciences, 564279), PE/Cy7 anti-mouse CD11c (N418, Biolegend, 117318), FITC anti-mouse CD80 (16-10A1, Biolegend, 104706), Percp/Cy5.5 anti-mouse CD86 (GL-1, Biolegend, 105028) and Brilliant Violet 421 anti-mouse I-A/I-E (M5/114.15.2, Biolegend, 107632). FITC anti-mouse CD3 (17A2, Biolegend, 100228), BUV661 anti-mouse CD4 (GK1.5, BD Biosciences, 612974), PE anti-mouse CD4(H129.19, Biolegend, 130310), BUV496 anti-mouse CD8a (53-6.7, BD Biosciences, 750024), PE/Cyanine7 anti-mouse CD8a (53-6.7, Biolegend, 100722), PE/Cy5 anti-mouse FOXP3 (FJK-16s, eBioscience™, 15-5773-80), Brilliant Violet 421 anti-mouse CD25 (A18246A , Biolegend, 113705), Brilliant Violet 605™ anti-mouse CD25 antibody (PC61, Biolegend, 102036),

BV421 anti-mouse Ki67 (16A8, Biolegend, 652411), BV421 anti-mouse FOXP3 (MF-14, Biolegend, 126419), APC anti-human/mouse Granzyme B (QA16A02, Biolegend, 372203), PE/Cyanine7 anti-mouse IFNγ (XMG1.2, Biolegend, 505826), Alexa Fluor® 647 anti-IDO1 (2E2/IDO1, Biolegend, 654003) and Zombie Aqua™ Fixable Viability Kit (Biolegend, 423102) were used for cell staining. For intracellular staining of FOXP3, Granzyme B, IFNγ, and Ki67, cells were fixed and permeabilized using a FOXP3/Transcription factor staining buffer set (eBioscience™, 00-5523-00) according to the manufacturer's protocol prior to antibody incubation.

Characterization

All reactions were performed in a dry atmosphere. Thin layer chromatography (TLC) was performed on silica gel 60 F254 precoated aluminum sheets (Merck) and visualized using fluorescence quenching. Chromatographic purification was performed using flash column chromatography on silica gel (neutral, Qingdao Haiyang Chemical Co., Ltd). ^{1}H nuclear magnetic resonance (NMR) spectra were recorded on a Bruker 400 spectrometer (Bruker BioSpin Corporation, USA) at 400 MHz in CDCl3 or DMSO-d6. Chemical shifts were calibrated to the residual solvent peak or tetramethylsilane (= 0 ppm). High-resolution mass spectrometry (HRMS)-ESI was performed using an AB TripleTOF 5600 plus System (AB SCIEX, Framingham, USA). UV absorption spectra were recorded with a UV-vis spectrometer (Shimadzu, UV-2700). Reverse-phase high performance liquid chromatography (RP-HPLC) was conducted using a Hitachi Chromaster 5000 system (Hitachi, Japan) with a YMC-Pack ODS-A column (5 μm, 250 × 4.6 mm). All HPLC runs used linear gradients of acetonitrile (solvent A) and water (solvent B) containing 0.1% trifluoroacetic acid (TFA, TCI, T0431).

Synthesis of MSA-2

Succinic anhydride (3.1 g, 30.9 mmol) and aluminum trichloride (2.7 g, 20.6 mmol) were dissolved in dichloromethane (DCM, 10 mL) and stirred at 0°C for 1 h. Subsequently, 5,6-dimethoxybenzo[b]thiophene (2.0 g, 10.3 mmol) dissolved in 40 mL of DCM was added dropwise to the reaction solution over 30 minutes. The reaction mixture was then stirred at 45°C for 6 hours. Upon confirming the completion of the reaction via TLC, the reaction mixture was poured into ice water and the solution pH was adjusted to 10 with sodium hydroxide. The filtrate obtained through filtration was further acidified to pH 2 with concentrated hydrochloric acid. The desired product precipitated as a

solid, which was collected by filtration. Finally, the precipitate was washed with water and DCM to yield a solid product (2.6 g, 85.6%).

Synthesis of MSA-2-SS-OH

To a solution of MSA-2 (200 mg, 0.68 mmol, 1.0 eq) in dry dichloromethane (DCM, 5 mL) was added 2,2'-DITHIODIETHANOL (157.2 mg, 1.0 mmol, 1.5 eq), 4-dimethylaminopyridine (DMAP) (166.2 mg, 1.4 mmol, 2.0 eq) and 1-(3-dimethylaminopropyl)-3-ethylcarbodiimide (EDC) (211 mg, 1.4 mmol, 2.0 eq). The reaction mixture was stirred at 45°C for 4 h and the solvent was removed by evaporation. The residue was dissolved in DCM and washed with 5% citric acid, saturated $NaHCO_3$ and brine. The organic layer was dried over anhydrous $Na2SO_4$, filtered, and evaporated under vacuum. The crude product was further purified by flash column chromatography on silica gel (DCM: methanol = 20:1) to afford the MSA-2-SS-OH as a pink solid (212 mg, 72.4%).

^1H NMR (400 MHz, CDCl3) δ 7.90 (s, 1H), 7.25 (s, 2H), 4.40 (t, J = 6.0 Hz, 2H), 3.98 (s, 3H), 3.96 (s, 3H), 3.90 (t, J = 8.0 Hz, 2H), 3.34 (t, J = 6.0 Hz, 2H), 2.94 (t, J = 6.0 Hz, 2H), 2.88 (t, J = 6.0 Hz, 2H), 2.81 (t, J = 6.6 Hz, 2H).

Synthesis of MSA-2 prodrug

To a solution of MSA-2-SS-OH (300 mg, 0.70 mmol, 1.2 eq) in dry dichloromethane (DCM, 5 mL) was added DHA (190.8 mg, 0.58 mmol, 1.0 eq), DMAP (106.2 mg, 0.87 mmol, 1.5 eq) and EDC (135 mg, 0.87 mmol, 1.5 eq). The reaction mixture was stirred at 45°C for 4 h and the solvent was removed by evaporation. The residue was dissolved in DCM and washed with 5% citric acid, saturated $NaHCO_3$ and brine. The organic layer was dried over anhydrous $Na2SO_4$, filtered, and evaporated under vacuum. The crude product was further purified by flash column chromatography on silica gel (DCM: methanol = 15:1) to afford the MSA-2-SS-OH as an orange solid (300 mg, 68%).

^1H NMR (400 MHz, CDCl3) δ 7.89 (s, 1H), 7.25 (s, 2H), 5.45 - 5.27 (m, 12H), 4.36 (dt, J = 14.0, 6.0 Hz, 4H), 3.98 (s, 3H), 3.96 (s, 3H), 3.33 (t, J = 6.0 Hz, 2H), 2.93 (td, J = 6.0, 4.0 Hz, 4H), 2.83 (m, 12H), 2.39 (d, J = 4.0 Hz, 4H), 2.07 (m, 2H), 0.97 (t, J = 8.0 Hz, 3H).

Synthesis of NLG919 prodrug

To a solution of NLG919 (160 mg, 0.57 mmol, 1.0 eq) in dry DCM (5 mL) was added DHA (223.2 mg, 0.70 mmol, 1.2 eq), DMAP (104 mg, 0.85 mmol, 1.5 eq) and EDC (132 mg, 0.85 mmol, 1.2 eq). The reaction mixture was stirred at 45°C overnight and the solvent was removed by evaporation. The residue was dissolved in DCM and washed with 5% citric acid, saturated $NaHCO_3$ and brine. The organic layer was dried over anhydrous $Na2SO_4$, filtered, and evaporated under vacuum. The crude product was further purified by flash column chromatography on silica gel (DCM: methanol = 10:1) to afford the NLG919 prodrug as an orange solid (188 mg, 61.7%).

^{1}H NMR (400 MHz, CDCl3) δ 7.71 (s, 1H), 7.54 (dd, J = 8.0, 4.0 Hz, 2H), 7.36 (t, J = 8.0 Hz , 1H), 7.24 (dd, J = 8.0, 2.0 Hz, 1H), 7.17 (s, 1H), 5.45 - 5.26 (m, 12H), 5.12 - 5.01 (m, 2H), 2.88 - 2.79 (m, 10H), 2.45 - 2.19 (m, 6H), 2.15 - 2.02 (m, 3H), 1.72 - 1.68 (m, 2H), 1.66 - 1.62 (m, 3H), 1.50 - 1.43 (m, 1H), 1.22 - 1.06 (m, 4H), 0.96 (t, J = 8.0 Hz, 3H).

Characterization of Nanoparticles

To assess particle size distribution and zeta potential, nanoparticles were diluted in PBS and analyzed using a Malvern Nano ZS instrument (Malvern Panalytical, UK). For transmission electron microscopy (TEM), particles were deposited onto a carbon film-coated 200-mesh copper grid (S7), stained with a 2% solution of uranyl acetate for 30 seconds, and observed using a 120 kV Transmission Electron Microscope (TEM, Tecnai G2 Spirit, Thermo FEI). Additionally, the sample was imaged on a Talos F200C microscope (200 kV, Thermo FEI).

Stability of nanoparticles

The stability of non-PEGylated nanoparticles and Immunomodulatory binary nanoparticles (iBINP) was evaluated in ddH_2O or PBS supplemented with 10% (v/v) fetal bovine serum (FBS) at 37°C. Dynamic light scattering analysis was employed to monitor changes in particle size and polydispersity index (PDI) for 7 days.

In vitro drug release kinetics of nanoparticles

The in vitro release of MSA-2 and NLG919, both in the presence and absence of Dithiothreitol (DTT, 10 mM) and porcine liver esterase (PLE, 15 U/L), was assessed via HPLC analysis. Nanoparticles containing approximately 5% drug loading (0.1 mg/mL MSA-2 and NLG919 equivalent concentration)

in phosphate-buffered saline (PBS) solutions were prepared, including PBS alone, PBS containing 10 mM DTT and 15 U/mL PLE. These nanoparticle solutions were then loaded into dialysis bags (Spectrum, molecular weight cutoff of 3.5 kDa) and dialyzed against PBS (pH 7.4) supplemented with 0.4% Tween-80 to simulate physiological conditions and facilitate drug release. The dialysis bags were incubated in an orbital shaking water bath at 37°C, with continuous shaking. At specified time intervals, aliquots of the releasing buffer were collected, and the total amount of released drugs was determined using analytical RP-HPLC. The RP-HPLC system employed acetonitrile/water as the mobile phase at a flow rate of 1.0 mL/min, with UV detection performed at wavelengths of 326 nm for MSA-2 and 220 nm for NLG919. The quantities of released compounds were calculated by generating standard curves.

Cytotoxic T Lymphocyte (CTL) Killing Assay

To generate antigen-specific CTLs, BMDCs from wild type C57BL/6 mice and splenocytes from OVA peptide (GenScript, RP10611) pulsed C57BL/6 mice were harvested and then cultured in the presence of 20 IU/mL of IL-2 (Abclonal, RP01384) for 2 days. The stimulated splenocytes and BMDCs were then co-cultured with B16F10-OVA cells at an effector-to-target ratio of 20:5:1. The BMDCs cells had been pre-treated with various drug formulations (STING-NP, IDO1-NP, FDC, or iBINP) for 24 h. After 48 h of co-culture, the cytotoxic activity was assessed. For the LDH release assay, the culture supernatant was collected, and LDH activity was measured using an LDH Cytotoxicity Assay Kit (Yeasen, 40209ES76) according to the manufacturer's protocol. The viability of the remaining tumor cells was evaluated using the CCK-8 assay (MCE, HY-K0301) and quantified by a microplate reader (Multiskan FC, Thermo Scientific) at 450 nm.

To precisely quantify cancer cell-specific death induced by CTLs, we employed a refined flow cytometry analysis. In this co-culture experiment, B16-OVA tumor cells were mixed with immune cells. To accurately distinguish between these two cell types and specifically assess the viability of the cancer cells, we utilized two key markers: CD45 and Propidium Iodide (PI). Samples were analyzed on a flow cytometer (CytoFlex LX, Beckman). Following gating on single cells, the tumor cell population was identified by gating on CD45-negative cells. The percentage of dead tumor cells was then quantified as the frequency of PI-positive cells within this CD45-negative gate.

Confocal Laser Scanning Microscopy (CLSM)

The cellular uptake and intracellular trafficking of nanoparticles were visualized by CLSM (Olympus, IX83-FV3000). MC38 cells or BMDCs were seeded onto glass-bottom dishes. The cells were then incubated with DiI-labeled iBINP (Yeasen, 710052ES10) at a final DiI concentration equivalent to 100 nM for 1, 4, or 8 h. After incubation, the cells were washed with PBS and stained with LysoTracker Green (Beyotime, C1047S) for 30 min to label lysosomes and Hoechst 33342 (Beyotime, C1029) for 15 min to label nuclei. After a final wash with PBS, the cells were imaged using a confocal laser scanning microscope. The red fluorescence signal represented nanoparticles, green for lysosomes, and blue for nuclei.

In vivo Imaging and Biodistribution Analysis

To evaluate the in vivo biodistribution and tumor-targeting capability of the nanoplatform, MC38 tumor-bearing C57BL/6 mice were injected intravenously with 100 μL of free Cy5.5 dye (lumiprobe, 670C0) or Cy5.5-labeled iBINP (at an equivalent Cy5.5 dose, 1 mg/kg) in PBS. In vivo near-infrared fluorescence (NIRF) imaging was performed at predetermined time points using an IVIS imaging system. At 24 hours post-injection, mice were euthanized, and major organs (heart, lungs, liver, spleen, kidneys) and tumors were excised for ex vivo imaging. Tissues were washed in 1× PBS and transferred to the imaging stage. Fluorescence was measured as average radiant efficiency, and regions of interest (ROIs) were drawn manually over the tumors and organs to quantify the signal using Living Image software.

Cellular Uptake Analysis by Flow Cytometry

To determine the cellular distribution of the nanoplatform within the tumor microenvironment and draining lymph nodes, tissues were harvested 24 hours post-injection. Tumors and lymph nodes were mechanically dissociated and enzymatically digested to obtain single-cell suspensions. The cells were then stained with a cocktail of fluorescently-labeled antibodies against surface markers, including CD45, CD3, F4/80, and CD11c. Cellular uptake of the Cy5.5-labeled nanoparticles was quantified by measuring the geometric mean fluorescence intensity (gMFI) of the Cy5.5 signal within specific cell populations using a flow cytometer (CytoFLEX LX, Beckman). The gating strategy involved first identifying live, single cells, followed by gating on CD45^{+} immune cells, which were further subdivided

into T cells ($CD3^+$), macrophages ($F4/80^+$), and dendritic cells (DCs; $CD11c^+$). $CD45^-$ cells were identified as tumor cells.

Evaluation of immunoregulatory activity in tumor-bearing mice

Mice were subcutaneously injected with MC38 cells (5×10^6 cells) into the right flank. Once tumors reached sizes of approximately 100 mm^3, the mice were randomly divided into five groups (n = 5-6 per group). Treatment consisted of three intravenous injections of STING-NP, IDO1-NP, and iBINP, each containing an MSA-2 dose of 30 mg/kg and NLG919 dose of 10 mg/kg, administered on days 0, 3, and 6. Free MSA-2/NLG919 was used for comparison. Following treatment, mice were euthanized, and tumors, spleens, blood, and tumor-draining lymph nodes (TDLNs) were collected. For flow cytometric analysis, tumors were dissociated using scissors and digested in a solution containing 10 U/mL Deoxyribonuclease I (DNase I, Sigma-Aldrich, 260913) and 1 mg/mL Collagenase IV (Sigma-Aldrich, C5138) in RPMI 1640 media for 30 min at 37 °C. After digestion, tissues were lysed, strained through a 70 μm cell strainer (Biosharp, BS-70-XBS), and diluted to a concentration of 2×10^7 cells/mL in PBS containing 2% fetal bovine serum (FBS) for staining with fluorescent antibodies. A volume of 100 μl of cell suspension for each flow test was transferred into a tube and treated with TruStain FcX (Biolegend, 101320) according to the manufacturer's instructions. Samples were then stained with several panels of antibodies, cells were washed twice, suspended in PBS, and analyzed using a CytoFlex LX flow cytometer, with data analyzed using CytExpert 2.5 software.

Online datasets analysis

To identify potential differentially expressed genes after STING agonists treatment, we screened genes that may be differentially expressed in mouse tumor models post treatments from the GEO datasets (GSE204825, GSE159825 and GSE134129). A total of 45 overlapped DEGs (FDR < 0.01, |logFC|≥ 1) were detected were identified by intersecting those datasets. For further data analysis, Gene set enrichment analysis (GSEA) GSEA was performed on the normalized data using the GSEA tool (http://www.broad.mit.edu/gsea/). The *P* values of the differences between the two gene sets were analyzed with the Kolmogorov–Smirnov test. Tumor Immune Estimation Resource (TIMER 2.0) was used to identify the correlation of the IDO pathway signature gene expression (*Ido1*, *Ido2*, *Tdo2*) with

immune infiltration level in COAD. GEO dataset (GSE112876) was used to analyze the cell specific response to IFNα and IFNγ in peripheral immunocyte populations in mice.

Western Blotting

Tumor or immune cells were lysed in RIPA buffer (Solarbio, R0010) supplemented with protease (Solarbio, A8260) and phosphatase inhibitors (Solarbio, P1260). Protein concentration was determined using a BCA protein assay kit (Beyotime, P0012). Equal amounts of protein (20-30 μg) per sample were separated by SDS-PAGE and transferred onto polyvinylidene difluoride (PVDF, Abclonal, RM00018) membranes. The membranes were blocked with 5% non-fat milk (Beyotime, P0216) in Tris-buffered saline (Solarbio, T1080) with Tween 20 (Servicebio, GC204002) for 1 h at room temperature, followed by incubation with primary antibodies overnight at 4°C. The primary antibodies used were against IDO1, TBK1, p-TBK1, IRF3, p-IRF3, and β-actin. After washing with TBST, the membranes were incubated with HRP-conjugated secondary antibodies for 1 h at room temperature. The protein bands were visualized using an enhanced chemiluminescence (ECL, Abclonal, RM00021P) detection system and imaged with a chemiluminescence imaging system (ChemiDoc MP, Bio-Rad).

RNA Extraction and Quantitative Real-time PCR (qPCR)

To evaluate the impact of each nanoparticle on the IFN-I response, we seeded 5 × 10^5 BMDCs per well in 12-well plates. Free drugs or different prodrugs were added at the indicated concentrations of MSA-2/NLG919 for 8 h of incubation. Total RNA from BMDCs was extracted using RNA isolater Total RNA Extraction Reagent (Vazyme, R401) and used to synthesize first-strand cDNA with HiScript II One Step RT-PCR Kit (Vazyme, P611) following the manufacturer’s instructions. Quantitative RT–PCR was performed using ChamQ Universal SYBR qPCR Master Mix (Vazyme, Q711) on a LightCycler 480 II (Roche). β-actin was employed as an endogenous normalization control to obtain relative expression data. The primer sequences are listed as follows:

Mouse *Ifnb1*	Forward	‘5-ATGAGTGGTGGTTGCAGGC-3’
	Reverse	‘5-TGACCTTTCAAATGCAGTAGATTCA-3’
Mouse *Ifng*	Forward	‘5-CAGCAACAGCAAGGCGAAAAAGG-3’
	Reverse	‘5-TTTCCGCTTCCTGAGGCTGGAT-3’

Mouse *Il6*	Forward	‘5-ACCAGAGGAAATTTTCAATAGGC-3‘
	Reverse	‘5-TGATGCACTTGCAGAAAACA-3’
Mouse *Tnf*	Forward	5-CCTGTAGCCCACGTCGTAG-3
	Reverse	5-GGGAGTAGACAAGGTACAACCC-3
Mouse *Actb*	Forward	‘5-ACACCCGCCACCAGTTCGC-3’
	Reverse	‘5-ATGGGGTACTTCAGGGTCAGGATA-3’
Mouse *Ido1*	Forward	‘5-TGGCGTATGTGTGGAACCG-3’
	Reverse	‘5-CTCGCAGTAGGGAACAGCAA-3’
Human *IFNB1*	Forward	‘5-ACACCCGCCACCAGTTCGC-3’
	Reverse	‘5-ATGGGGTACTTCAGGGTCAGGATA-3’
Human *IFNG*	Forward	‘5-ATGACCAACAAGTGTCTCCTCC-3’
	Reverse	‘5-GGAATCCAAGCAAGTTGTAGCTC-3’
Human *IL6*	Forward	‘5-ACTCACCTCTTCAGAACGAATTG-3’
	Reverse	‘5-CCATCTTTGGAAGGTTCAGGTTG-3’
Human *TNF*	Forward	‘5-CCTCTCTCTAATCAGCCCTCTG-3’
	Reverse	‘5-GAGGACCTGGGAGTAGATGAG-3’
Human *ACTB*	Forward	‘5-CATGTACGTTGCTATCCAGGC-3’
	Reverse	‘5-CTCCTTAATGTCACGCACGAT-3’
Human *IDO1*	Forward	‘5-GCCAGCTTCGAGAAAGAGTTG-3’
	Reverse	‘5-ATCCCAGAACTAGACGTGCAA-3’

Enzyme-Linked Immunosorbent Assay (ELISA)

The concentrations of various cytokines, including IFNβ (NOVUSBIO, VAL612), IFNγ (NOVUSBIO, VAL607), IL6 (NOVUSBIO, VAL604G), and TNFα (NOVUSBIO, VAL609), in cell culture supernatants or mouse serum were quantified using commercial ELISA kits according to the manufacturer's instructions. Briefly, 96-well plates were coated with capture antibodies overnight. After washing and blocking, samples and standards were added to the wells and incubated for 2 h. Following another wash step, detection antibodies were added. Finally, after incubation with streptavidin-HRP and TMB substrate, the reaction was stopped, and the absorbance at 450 nm was measured using a microplate

reader (Multiskan FC, Thermo Scientific). Cytokine concentrations were calculated based on the standard curve.

Histological analysis and immunostaining of paraffin-embedded sections

For histological analysis, the excised tumors and organs were fixed with 4% paraformaldehyde in phosphate buffer (Solarbio, P1110), embedded in paraffin, and sectioned into 5 μm thick slices. The tumor and organ sections were stained with H&E. For immunohistochemical analysis, the paraffin-embedded sections were first dewaxed followed by antigen retrieval. The slices were then placed in a 3% solution of hydrogen peroxide and incubated at room temperature away from light for 25 min. The slides were then placed in phosphate-buffered saline (PBS, pH 7.4) and subjected to three washes of 5 min each on a decolorizing shaking table. The slides were then placed in a 3% solution of bovine serum albumin for 25 min and subsequently incubated with the primary antibodies anti-FOXP3 (Servicebio, GB112325, 1:200) and anti-IDO1 (Abclonal, A12125, 1:200) in a wet box overnight at 4°C. Finally, the slices were counterstained with hematoxylin, dehydrated using an ethanol gradient, cleared using xylene, and finally, mounted in a mounting medium and a cover slip placed. To assess the infiltration of immune cells, the sections were then incubated with anti-CD8a (Cell signaling technology, 98941, 1:200), anti-Granzyme B (Servicebio, GB15092, 1:200), anti-FOXP3 (Servicebio, GB112325, 1:200), and anti-IDO1 (Abclonal, A1614, 1:200) antibodies overnight at 4°C, followed by washes with PBS and labeling with the secondary antibodies Alexa Fluor 488-Labeled Goat Anti-Rabbit IgG (Servicebio, GB25303, 1: 300) or CY3-tagged Goat anti-mouse IgG (Servicebio, GB21303, 1: 300). After washing with PBS, the sections were mounted with 4′,6-diamidino-2-phenylindole (DAPI, Servicebio, G1012). Images were acquired at the indicated resolutions using an Olympus VS120 Virtual Slide System.

Generation of Ido1-Overexpressing and Knockout Cell Lines

To generate the IDO1-overexpressing MC38 cell line (MC38^{Ido1}), the pLV3-CMV-*Ido1*(mouse)-Puro plasmid was purchased from HZREPOBIO (Hangzhou, China). Lentiviral particles were produced by transfecting HEK293T cells and subsequently used to transduce MC38 cells. Following selection with puromycin, stable single-cell clones were isolated and expanded. Successful overexpression of *Ido1*

was confirmed by both qPCR and Western Blot analysis. The 4T1 Ido1-knockout cell line (4T1$^{Ido1\text{-}KO}$) was commercially constructed by Ubigene Biosciences Co., Ltd. Briefly, the cell line was generated using the CRISPR/Cas9 system, where ribonucleoprotein (RNP) complexes containing Cas9 and a single guide RNA (sgRNA) targeting a key exon of the *Ido1* gene were delivered into 4T1 cells via electroporation. After selection, single-cell clones were obtained by the limited dilution method, and successful knockout was validated by PCR and genomic DNA sequencing. The sgRNA and primers used for CRISPR/Cas9 editing here are designed as G1: TAGGGAACAGCAATATTGCG GGG; Forward: CTAGCTAACATGGTGTCCTGTG, Reverse: GTGCTGCAAGTTCGTTGGCC.
