# Supplementary material for: Targeting STING-induced immune evasion with nanoparticulate binary pharmacology improves tumor control in mice
Source: J Clin Invest. 2025 Oct 23;135(24):e192397. doi: 10.1172/JCI192397 (PMC12700560; doi:10.1172/JCI192397)

Full unedited blot for Figure 2K

Figure 2K

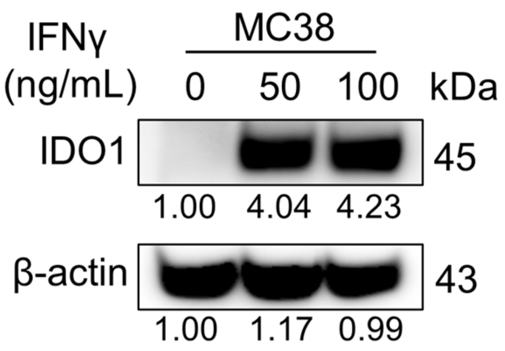

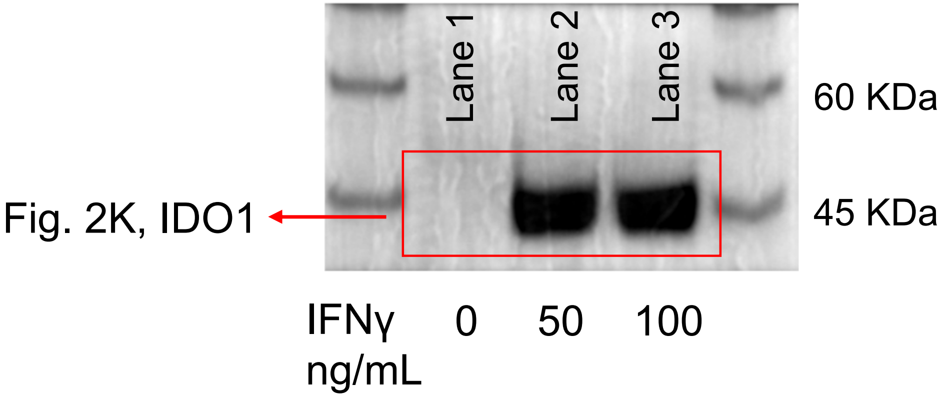

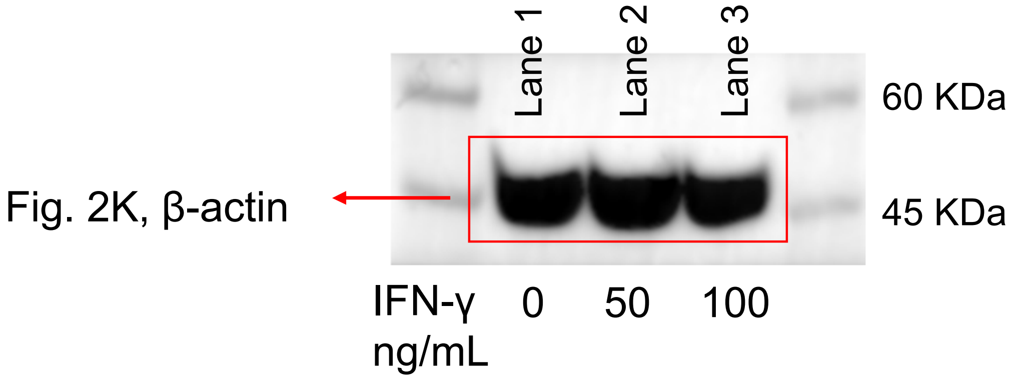

Full unedited blot for Figure 2N

Figure 2N

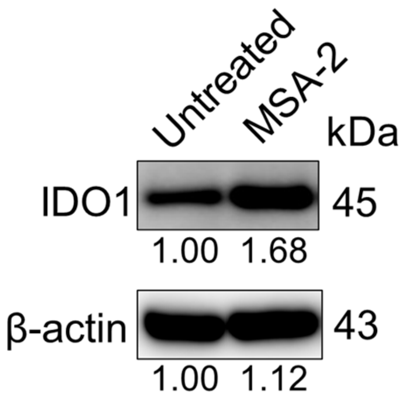

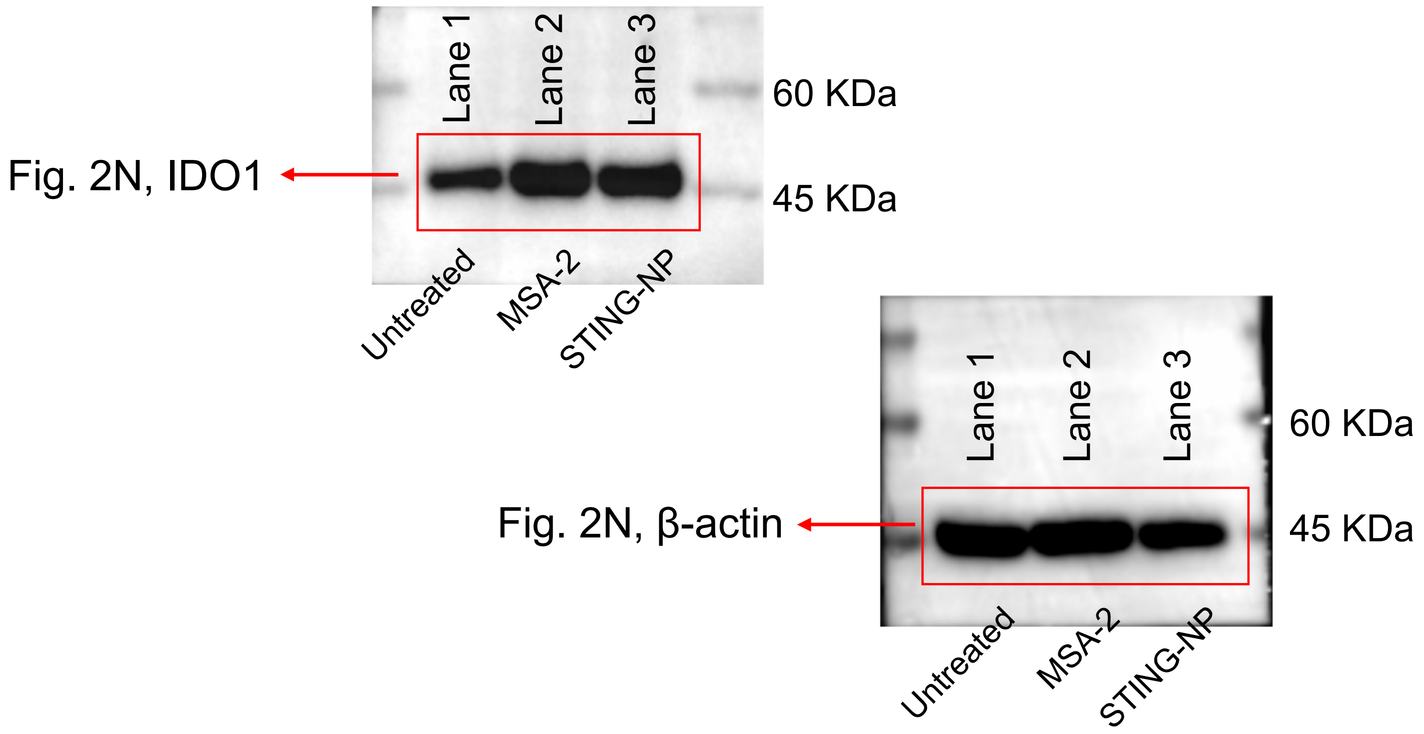

Full, uncropped blot related to Figure 2N. The blot shows the full image for the western blot presented in Figure 2N. The lane 3, corresponding to the STING-NP treatment group, was not included in the main figure to maintain narrative consistency with the main text, as the STING-NP group is introduced later in the manuscript.

Figure 4G

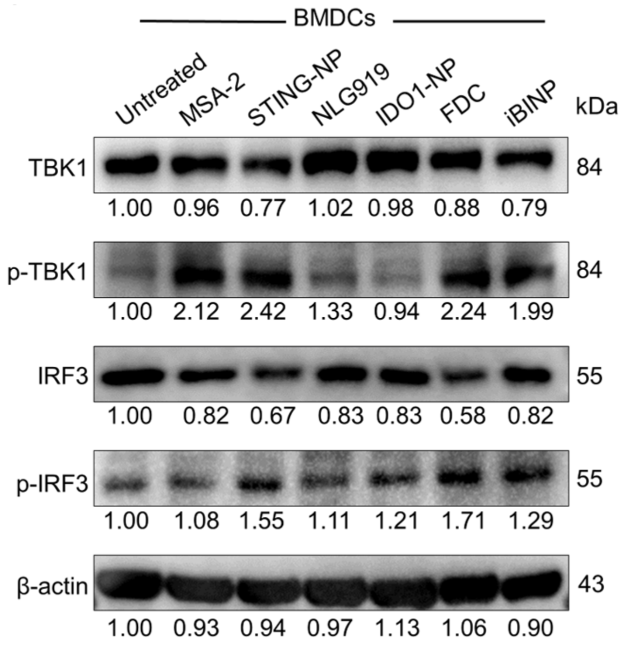

Full unedited blot for Figure 4G part 1

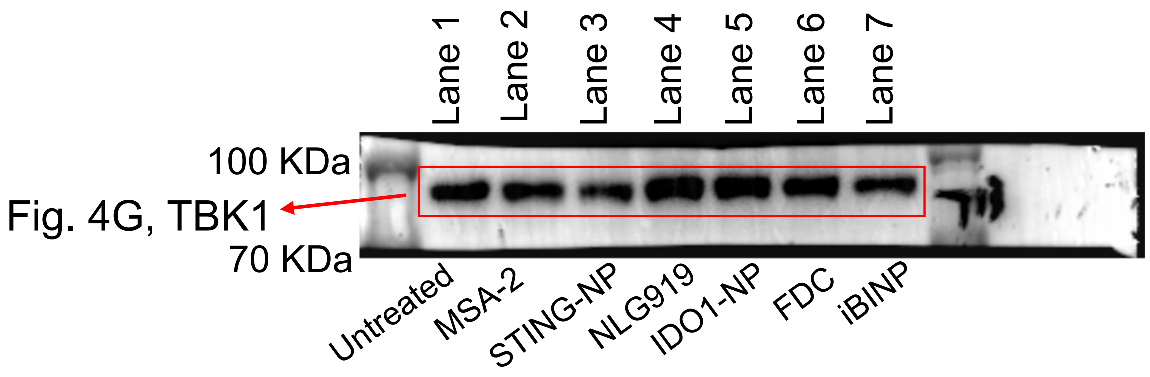

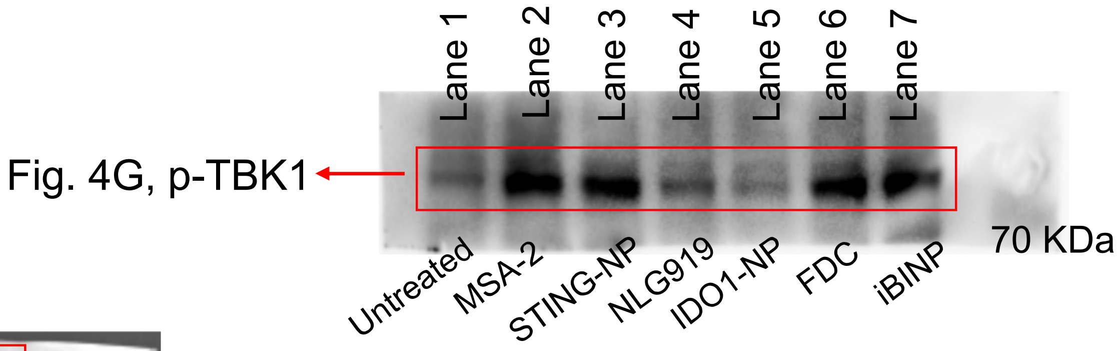

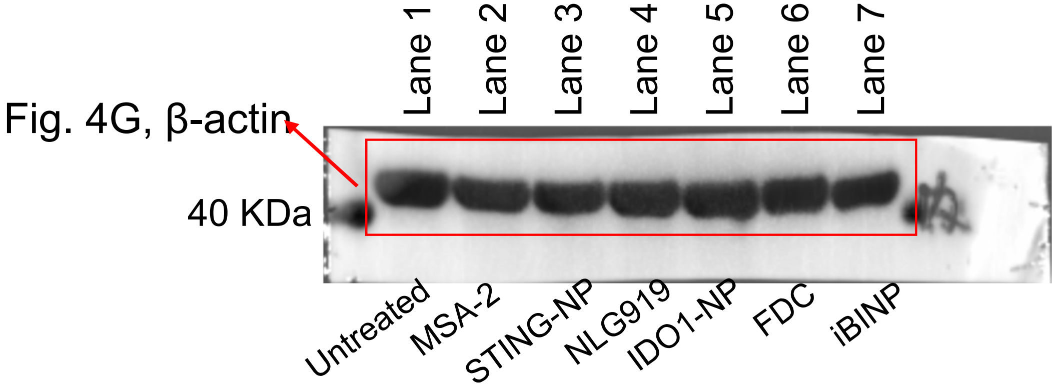

Full unedited blot for Figure 4G part 2

Figure 4G

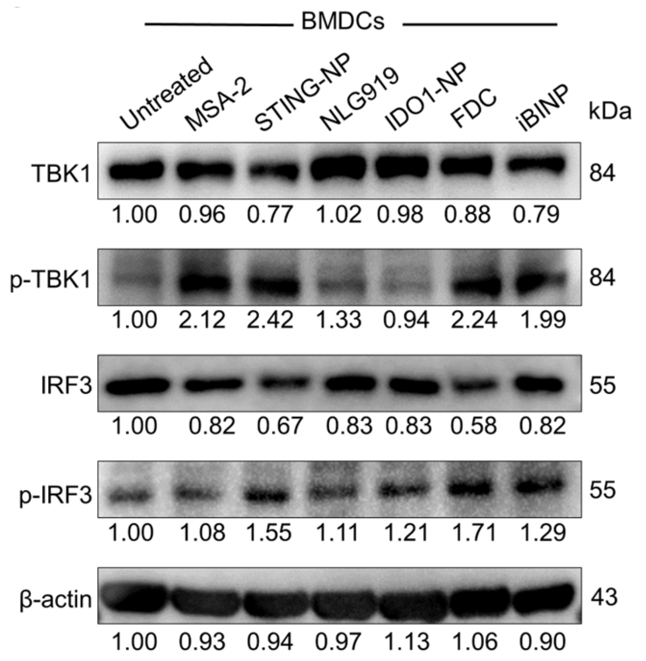

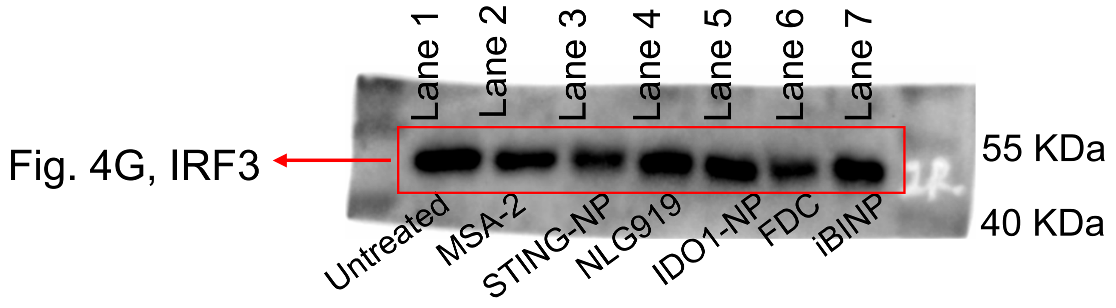

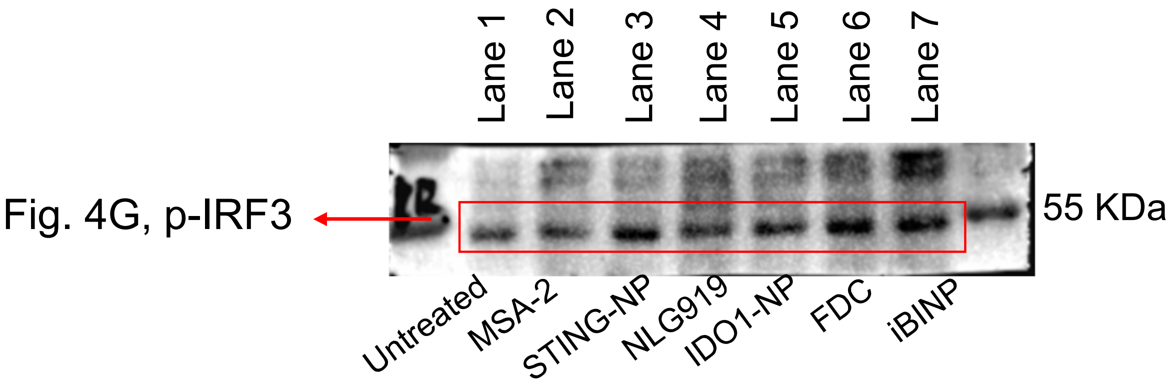

Full unedited blot for Figure 6K

Figure 6K

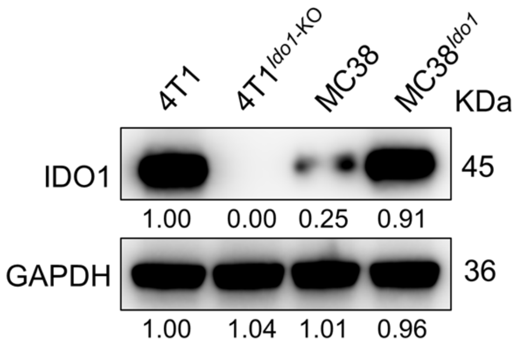

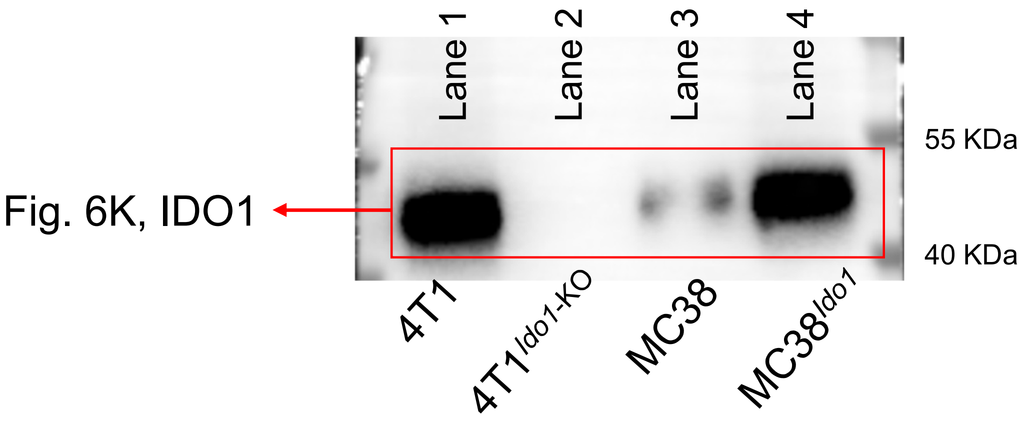

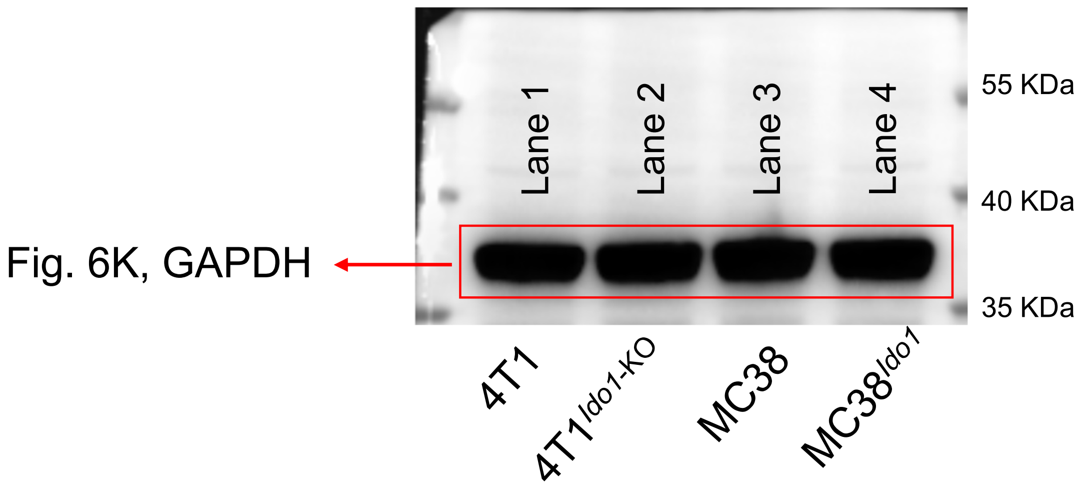

Supplement: Unedited blot and gel images [file jci-135-192397-s010.pdf]
